# Negative regulation of TGF-β1-induced MKK6-p38 and MEK-ERK signalling and epithelial-mesenchymal transition by Rac1b

**DOI:** 10.1038/s41598-017-15170-6

**Published:** 2017-12-11

**Authors:** David Witte, Hannah Otterbein, Maria Förster, Klaudia Giehl, Robert Zeiser, Hendrik Lehnert, Hendrik Ungefroren

**Affiliations:** 1 First Department of Medicine, University Hospital Schleswig-Holstein (UKSH), Campus Lübeck, and University of Lübeck, 23538 Lübeck, Germany; 2 Signal Transduction of Cellular Motility, Internal Medicine V, Justus-Liebig-University Giessen, 35392 Giessen, Germany; 3 Department of Hematology and Oncology, Freiburg University Medical Center, Albert-Ludwigs-University, 79106 Freiburg i.Br., Germany; 4 Department of General and Thoracic Surgery, UKSH, Campus Kiel, 24105 Kiel, Germany

## Abstract

Prompted by earlier findings that the Rac1-related isoform Rac1b inhibits transforming growth factor (TGF)-β1-induced canonical Smad signalling, we studied here whether Rac1b also impacts TGF-β1-dependent non-Smad signalling such as the MKK6-p38 and MEK-ERK mitogen-activated protein kinase (MAPK) pathways and epithelial-mesenchymal transition (EMT). Transient depletion of Rac1b protein in pancreatic cancer cells by RNA interference increased the extent and duration of TGF-β1-induced phosphorylation of p38 MAPK in a Smad4-independent manner. Rac1b depletion also strongly increased basal ERK activation - independent of the kinase function of the TGF-β type I receptor ALK5 - and sensitised cells towards further upregulation of phospho-ERK levels by TGF-β1, while ectopic overexpression of Rac1b had the reverse effect. Rac1b depletion increased an EMT phenotype as evidenced by cell morphology, gene expression of EMT markers, cell migration and growth inhibition. Inhibition of MKK6-p38 or MEK-ERK signalling partially relieved the Rac1b depletion-dependent increase in TGF-β1-induced gene expression and cell migration. Rac1b depletion also enhanced TGF-β1 autoinduction of crucial TGF-β pathway components and decreased that of TGF-β pathway inhibitors. Our results show that Rac1b antagonises TGF-β1-dependent EMT by inhibiting MKK6-p38 and MEK-ERK signalling and by controlling gene expression in a way that favors attenuation of TGF-β signalling.

## Introduction

Pancreatic ductal adenocarcinoma (PDAC) is one of the most deadliest diseases for which no curative therapies are available to date. To successfully establish prevention and treatment strategies for this disease, a better understanding of the molecular events underlying PDAC tumourigenesis is mandatory. Transgenic mouse models have shown that aggressive PDAC develops after pancreas-specific inhibition of transforming growth factor-beta (TGF-β) signalling in cooperation with active K-Ras expression^1^. However, the effector pathways of the TGF-β/K-Ras crosstalk remain elusive. Data from a *K-ras* ^G12D^ murine model with pancreas-specific ablation of *RAC1* suggested that the protein product(s) of *RAC1* is a crucial mediator of TGF-β/K-Ras-driven tumourigenesis since it prevented tumour development and significantly prolonged survival in these mice^2^. Although the oncogenic role of *Rac1* in this context has clearly been established, data interpretation remains problematic as *Rac1* gives rise to two different proteins, Rac1 and its splice variant, Rac1b. Rac1b differs from Rac1 by inclusion of a short exon (exon 3b, comprising 19 amino acids) close to the switch II region^3,4^. As a consequence of this modification, Rac1b has been found to have an accelerated GDP/GTP exchange and delayed GTP hydrolysis^5^ and to differ from Rac1 in certain signalling and functional properties. Rac1b does not interact with RhoGDI or p21-activated kinase and does not induce lamellipodia formation^6^, but retains the potential to increase cellular reactive oxygen species^7^. Since Rac1b is expressed at a much lower level than Rac1 in cells, it is normally not detected in immunoblot analyses and thus not analysed. Moreover, because of inevitable co-deletion of Rac1b upon *RAC1* ablation, the antitumour effects observed in the above mentioned mouse model cannot be ascribed unequivocally to the absence of Rac1. A solution to this dilemma would be a selective depletion of exclusively one of both isoforms, however, such data are not yet available. As far as Rac1 is concerned, we have shown earlier that Rac1 promotes TGF-β1 signalling in PDAC-derived cell lines towards a pro-metastatic outcome by enhancing TGF-β1-induced Smad2 activation, epithelial-mesenchymal transition (EMT), and random cell migration and invasion^8^.

Recently, we have detected Rac1b protein in tumour tissues of PDAC patients with expression being most prominent in the tumour cell fraction. Intriguingly, high Rac1b expression correlated with fewer metastases and significantly prolonged survival times compared to patients that lacked Rac1b expression in their tumour cells^9^. These finding argue in favor of an antimetastatic - and thus Rac1 antagonistic - effect of Rac1b in the context of a TGF-β1-rich microenvironment. It was therefore of interest to study *in vitro* i) how Rac1b controls tumour cell responses to TGF-β that are associated with malignant conversion such as EMT and cell migration/invasion and ii) which signalling pathways are targetted by Rac1b. In keeping with the idea that Rac1b represents an endogenous inhibitor of Rac1, we observed earlier that Rac1b inhibits TGF-β1-induced random cell migration and suppresses the C-terminal phosphorylation, and thus activation, of both Smad2 and Smad3^9^. TGF-β-induced activation of Smad complexes has crucial roles during induction of EMT^10,11^. However, whereas Smad4 and Smad3 promote EMT, Smad2 can inhibit it^12^. Hence, negative regulation of Smad2 *and* Smad3 activation would not explain the effect, if any, of Rac1b on TGF-β-induced EMT.

Various studies have shown that TGF-β1-dependent control of EMT and mesenchymal traits such as matrix production and cell motility may not only depend on canonical Smad- but also on non-canonical Smad and non-Smad signalling, sometimes in a tissue and cell-type specific manner^13–15^. Non-Smad signalling during EMT leads to activation of Rho GTPases^16^, mitogen-activated protein kinase (MAPK) pathways, and the PI3 kinase-Akt-mTOR pathway^13–15^. The MKK3/6-p38^10,11,13,17^ and the MEK-extracellular signal-regulated kinase (ERK) MAPK pathways^10,11,14,18^ control non-transcription changes/gene reprogramming and during EMT cooperate with Smad-mediated gene expression, *e.g*. through the transcription factor ATF2^19^, but may also directly regulate the stabilities and activities of Smads^15^. The ubiquitin ligase TRAF6 binds to the TGF-β type I receptor ALK5 and mediates Smad-independent activation of the MAPKKK TAK1, the MAPKKs MKK3/MKK6, and the JNK/p38 MAPKs^20^. The ERK pathway is stimulated by activated ALK5 through tyrosine phosphorylation of the adaptor protein Shc, allowing docking of the Grb2-Sos1 complex which subsequently leads to downstream activation of the Ras-Raf-MEK-ERK pathway^21^.

In light of the previously observed (negative) regulation of TGF-β1-induced Smad2/3 activation and random cell migration in PDAC-derived cells^8,9^, it is conceivable that Rac1b also affects activity of TGF-β1-dependent MKK6-p38 and/or MEK-ERK signalling as well as adoption of a mesenchymal phenotype. It should be mentioned, however, that due to the Smad- and ALK5 serine/threonine kinase-independent activation of p38 and ERK1/2, respectively, the effects of Rac1b on the Smad signalling pathway are not predictive of the effects of Rac1b on either the MKK6-p38 or the MEK-ERK signalling pathway. To study the effect of Rac1b on TGF-β1-induced p38 and ERK activation and on EMT, we primarily utilized the human PDAC-derived and TGF-β1-responsive cell lines Panc1, Colo357 and IMIM-PC1. In some experiments we included non-tumourigenic cells of pancreatic and non-pancreatic origin to evaluate whether the observed effects were tumour and tissue-specific, respectively.

## Methods

### Antibodies and Reagents

The following primary antibodies were used: Anti-phospho-p38 (#9211), anti-p38 (#9212), anti-phospho-ERK1/2 (#4370), anti-E-Cadherin (#3195), anti-Snail (#4719) (all from Cell Signaling Technology, Frankfurt/Main, Germany), anti-HSP90 (both #sc-7947 and #sc-13119), anti-MKK6 (sc-6073), anti-TGF-β receptor I/ALK5 (V22, #sc-398), anti-TGF-β1 (3C11, #sc-130348) (all from Santa Cruz Biotechnology, Heidelberg, Germany), anti-ERK1/2 (#AF1576, R&D Systems, Wiesbaden, Germany) anti-Rac1b (#09-271, Merck Millipore, Darmstadt, Germany), anti-Rac1 (#610650), anti-Cip1/WAF1 (#610233) (both from BD Biosciences, Heidelberg, Germany), anti-β-actin (#A1978, Sigma, Deisenhofen, Germany). anti-Flag M2 (F3165, Sigma), HRP-linked anti-rabbit (#7074), anti-mouse (#7076) and anti-rat (#7077) secondary antibodies were from Cell Signaling Technology, anti-goat secondary antibody (#ab6741) was from Abcam (Cambridge, UK). Recombinant human (rh) TGF-β1 (#300-023) was purchased from ReliaTech (Wolfenbüttel, Germany) and used at a concentration of 5 ng/ml. The p38 inhibitor SB203580, the MEK1 inhibitor U0126 and the ALK5 inhibitor SB431542 were purchased from Calbiochem and used at a concentration of 10 μM (SB203580, UO126) and 5 μM (SB431542). Treatment of cells with these inhibitors for up to 48 h had no gross effect on cell viability.

### Cell Culture and Generation of Panc1 Cells Ectopically Expressing HA-Rac1b or Dominant-negative Mutants of MKK6 or ALK5

Panc1 and Colo357 human PDAC cells were originally obtained from ATCC (Manassas, VA). Another PDAC-derived cell line, IMIM-PC1, was obtained from P. Real (University of Madrid) and kindly supplied by A. Menke (University of Giessen). Panc1 and Colo357 cells were cultured in RPMI 1640 supplemented with 10% heat-inactivated fetal bovine serum (FBS), 1% Penicillin-Streptomycin-Glutamine (Life Technologies) and 1% sodium pyruvate (Merck Millipore). IMIM-PC1 cells and HaCaT immortalized human keratinocytes (ATCC) were maintained in DMEM containing the same supplements. The human ductal pancreatic epithelial cell line HPDE6c7 was a kind gift of S. Sebens (University of Kiel) and was cultured as described^22^. The generation of Panc1 cell clones ectopically expressing HA-Rac1b^9^, dominant-negative mutants of MKK6 (MKK6_KA_, carrying K82A substitution)^23,24^, ALK5 (ALK5_KR_, carrying K232R substitution)^25^ or Flag-Smad4_1-514_^26^ was described in detail earlier. In all cases, cells were stably transduced using the retroviral vector TJBA5bMoLink-neo, followed by selection of successfully transduced cells with G418 (700 µg/ml) and generation of individual cell clones using limited dilution. Pooled empty-vector transductants served as a control. Ectopic expression of the mutant proteins was verified by immunoblotting for Rac1b, MKK6 and ALK5, respectively.

### Cell Counting

The number of Panc1 cells with spindle-shaped morphology after Rac1b siRNA transfection and TGF-β1 treatment was counted per visual field by two investigators in a blinded fashion.

### Quantitative real-time RT-PCR (qPCR) Analysis

Total RNA was extracted from Panc1 cells grown in 24-well plates using PeqGold RNAPure (Peqlab, Erlangen, Germany) and purified according to manufacturer’s instructions. For each sample, 2.5 μg RNA were subjected to reverse transcription for 1 h at 37 °C, using 200 U M-MLV Reverse Transcriptase and 2.5 μM random hexamers (both from Life Technologies) in a total volume of 20 μl. Relative mRNA expression of target genes was quantified by real-time PCR on an I-Cycler (BioRad) using Maxima SYBR Green Mastermix (Thermo Fisher Scientific). Data were normalized to the expression of TATA-box-binding protein (TBP) for each sample. For PCR primers see Supplementary Table S1.

### Transient Transfection of siRNAs

On day 1 and 2 after seeding into 6, 12 or 24-well plates (Nunclon^TM^ Delta Surface, Nunc, Roskilde, Denmark) cells were transfected with 50 nM of siRNA specific for Rac1b or scrambled control, siRNA specific for Rac1 + Rac1b (ON-TARGETplus SMARTpool, a mixture of four prevalidated siRNAs) or matched negative control (non-target control SMARTpool), both purchased from GE Healthcare Dharmacon (Epsom, UK) or a pool of three different and validated siRNAs to ALK5 (Validated Stealth RNAi^TM^ siRNA (Set of 3) HSS110695, HSS110696, HSS110697) or matched negative control (Life Technologies), or siRNA to BGN or matched negative control (Qiagen, CA) for 4 h using Lipofectamine RNAiMAX (Life Technologies) at a concentration of 0.5%.

### Reporter gene Assay

For reporter gene assays, Panc1 cells were seeded in 96-well plates (Nunclon^TM^ Delta Surface) and cotransfected on the following day serum-free for 4 h with Lipofectamine 2000 (Life Technologies) and 50 nM of BGN siRNA or control siRNA. Afterwards, cells received standard growth medium. Twenty-four h after the start of the first transfection, cells underwent a second round of transfection with 50 nM each of BGN or control siRNA plus 100 ng/well of the TGF-β-responsive luciferase reporter plasmid p3TP-Lux and 25 ng/ml pRL-TK-Luc, a vector encoding Renilla luciferase (Promega, Heidelberg, Germany). On the next day, cells were stimulated with 5 ng/ml TGF-β1 for 24 h and then lysed in Glo lysis buffer (Promega) and subjected to dual luciferase measurement with the Dual Luciferase Assay System according to the manufacturer’s protocol (Promega).

### [^3^H]-Thymidine Incorporation Assay

This assay was performed exactly as described in detail earlier^8^.

### Cell Lysis and Immunoblotting

Confluent cells were washed once with ice-cold PBS and lysed with 1x PhosphoSafe lysis buffer (Merck Millipore). Cell lysates were sonicated and centrifuged for 10 min at 14.000 × g and 4 °C following determination of total protein concentration in supernatants using BioRad DC Protein Assay. Samples containing equal amounts of protein were prepared using 3x SDS Sample Buffer and 125 mM Dithiothreitol (both from New England Biolabs), subjected to gel electrophoresis using BioRad mini-PROTEAN TGX any-kD precast gels and blotted to 0.45 μm PVDF membranes. Membranes were blocked with nonfat dry milk (Carl Roth GmbH, Karlsruhe, Germany) or BSA (Sigma-Aldrich) and incubated with primary antibodies at 4 °C overnight. HRP-linked secondary antibodies and Amersham ECL Prime Detection Reagent (GE Healthcare) were used for detection of proteins on a BioRad ChemiDoc XRS imaging system. RotiFree stripping buffer (Carl Roth GmbH) was used for membrane stripping. Signal intensities were quantified by densitometry and computed with either NIH image J or Image Lab (version 5.2.1, BioRad).

### ELISA for TGF-β1

Twenty-four h after the second transfection with either control siRNA or TGF-β1 siRNA Panc1 cells received fresh medium containing 0.5% FBS and culture supernatants were allowed to be conditioned for another 24 h. Aliquots from the culture supernatants were cleared by centrifugation, appropriately diluted and subjected to a TGF-β1-specific ELISA (Human/Mouse TGF beta1 ELISA Ready-SET-Go!, eBioscience/Affymetrix Inc. San Diego, CA) according to the manufacturer’s instructions. The detection limit was 25 pg/ml. Data for total TGF-β1 were normalised to the cell number from the respective well.

### Real-time Cell Migration Assays

The xCELLigence® DP system (ACEA Biosciences, distributed by OLS, Bremen, Germany) was used to measure random migratory activity of wild-type Panc1 cells and stably transduced Panc1 cell clones. Cells were seeded in 6-well plates, treated as desired and then serum-starved (standard growth medium containing 0.5% FBS) for 24 h prior to transferring the cells to the assay. For all assays, RPMI with 1% FBS and a final concentration of TGF-β1 of 5 ng/ml in both the upper and lower chambers of the CIM plates-16 was used. CIM plates-16 were prepared according to the instruction manual and previous descriptions^9,27,28^. The underside of the upper chambers of the CIM plate-16 was coated with 30 μl of collagen I (400 μg/ml) and allowed to dry for at least 2 h prior to plate assembly. 40,000 cells were loaded into each well of the upper chamber immediately after addition of TGF-β1 to the cell suspensions. Data acquisition was at 15 min intervals and analysis done with the RTCA software.

### Statistical Analysis

Statistical significance was calculated using the Mann-Whitney u test. Results were considered significant at *p* < 0.05 (*). Higher levels of significance were *p* < 0.01 (**) and *p* < 0.001 (***).

### Data Availability Statement

All data generated or analysed during this study are included in this published article (and its Supplementary Information files).

## Results

### Rac1b silencing increases the duration of the p38 MAPK phosphorylation response to TGF-β1

**Figure 1:**
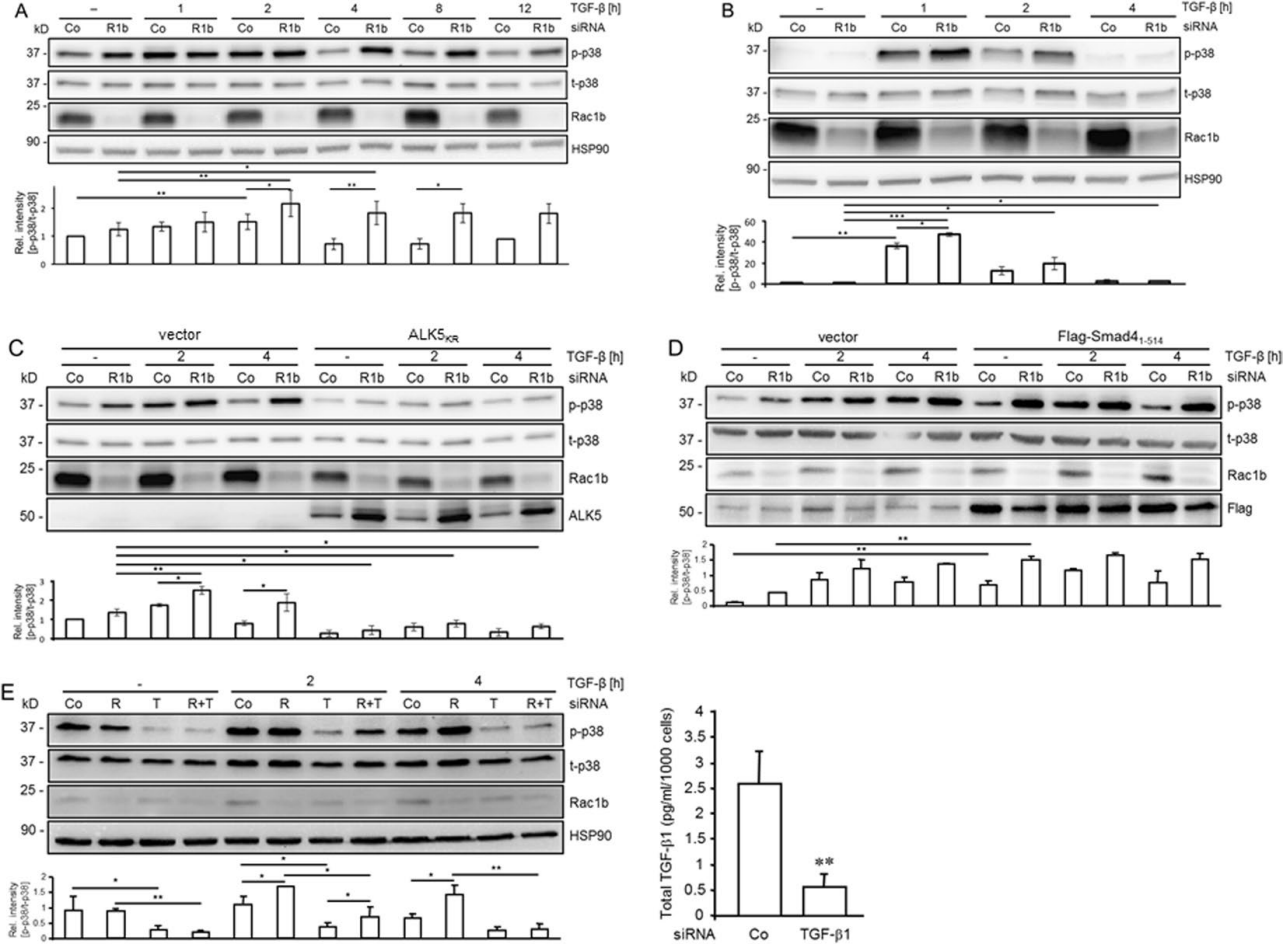
Depletion of Rac1b increases TGF-β1-induced p38 MAPK activation. (**A**) Panc1 cells were transiently transfected with 50 nM each of a specific Rac1b siRNA (R1b) or an appropriate control siRNA (Co). Transfected cells were serum-starved for 24 h, treated with TGF-β1 for various times as indicated and subjected to immunoblotting for phosphorylated p38 MAPK (p-p38) and total p38 MAPK (t-p38) as well as HSP90 as a control for equal loading. Detection of Rac1b on the same membrane served as a control for transfection efficiency. The chart below shows the relative values for mean intensity ± SD of the bands for p-p38 normalized to those of t-p38 from six (time points 0, 2 and 4 h) or three (time points 1, 8, and 12 h) independent experiments. Asterisks indicate a significant difference in p-p38 band intensity between the indicated samples. (**B**) As in (**A**), except that HaCaT cells were used. (**C**) Panc1 cells stably expressing empty retroviral vector or ALK5_KR_ were transiently transfected with 50 nM each of a siRNA specific for Rac1b or an irrelevant control siRNA and subjected to immunoblotting for p-p38, t-p38, Rac1b, ALK5, and HSP90 as a loading control. The chart below the blots shows the relative intensity of the bands for p-p38 normalized to t-p38 from three independent experiments (mean ± SD, n = 3). Asterisks indicate a significant difference. (**D**) Panc1 cells stably expressing empty retroviral vector or Flag-Smad4_1-514_ were transiently transfected with 50 nM each of a siRNA specific for Rac1b or an irrelevant control siRNA and immunoblotted for p-p38, Rac1b, t-p38, Flag, and HSP90 as a loading control. The chart below the blots shows the relative intensity of the bands for p-p38 normalized to t-p38 from three independent experiments (mean ± SD, n = 3). Asterisks indicate a significant difference. Data displayed in each panel are from the same blot/gel treated subsequently with antibodies to p-p38, Rac1b, HSP90, and either ALK5 or Flag, then stripped and reincubated with an antibody to t-p38. (**E**) Panc1 cells were transiently transfected with 50 nM of a Co siRNA, Rac1b siRNA (R), TGF-β1 siRNA (T) or a combination of Rac1b siRNA and TGF-β1 siRNA (R + T) as indicated and further processed as described in (A). The chart below the blots shows the relative values for mean intensity ± SD of the bands for p-p38 normalized to those of t-p38 from three independent experiments. Asterisks indicate a significant difference in p-p38 band intensity between the indicated samples. The graph on the right-hand side of the blots depicts the concentration of total TGF-β1 in culture supernatants (conditioned for 24 h) from Co siRNA and TGF-β1 siRNA transfected Panc1 cells as determined by ELISA (mean ± SD, n = 3, *p* < 0.01).

We have previously shown that TGF-β1-induced activation of both Smad2 and p38 MAPK is Rac1-dependent in PDAC-derived cells^8,29^. Since Rac1b inhibits Smad2/3 activation, we hypothesised that Rac1b might also inhibit p38 activation. To study this in more detail, we first depleted the PDAC cell lines Panc1 and Colo357 of cellular Rac1b protein by transfection of a siRNA targetting exon 3b unique to Rac1b^9^, or an irrelevant control siRNA, and subsequently exposed the cells for various times to rhTGF-β1. In Panc1 cells, the levels of phospho-p38 (p-p38) increased in both control siRNA and Rac1b siRNA-transfected cells and in both groups the differences became significant at 2 h of TGF-β1 treatment (Fig. 1A). At the 4 h time point p-p38 levels in control siRNA-transfected cells had returned to baseline levels while those of Rac1b siRNA-transfected cells remained elevated for up to 12 h (Fig. 1A). A very similar and delayed activation of p38 by TGF-β with peak levels of p-p38 at 2 h and a subsequent decline has been described earlier in (non-transfected) Panc1 cells^30^. In Colo357 cells, p-p38 levels peaked at 1 h of TGF-β1 treatment in both control siRNA and Rac1b siRNA-transfected cells and declined thereafter but remained elevated over those in non-TGF-β1-treated controls (Supplementary Fig. S1). As in Panc1 cells, the p-p38 levels in Rac1b-depleted at all time points appeared higher than in control cells, however, only after 4 and 8 h of TGF-β1 stimulation these differences reached statistical significance (Supplementary Fig. S1).

To analyse whether or not the Rac1b effect was specific for PDAC-derived cells, we studied the effects of Rac1b depletion in HaCaT keratinocytes (Fig. 1B). Upon an 1-h TGF-β1 treatment, p-p38 levels in both control siRNA and Rac1b siRNA-transfected cells rose dramatically and thereafter declined but for Rac1b siRNA remained elevated over non-stimulated controls even after 4 h of TGF-β1 exposure (Fig. 1B, lane 2 *vs*. 8). After 1 h but not after 2 and 4 h, the TGF-β1 stimulated levels of p-p38 were significantly higher in Rac1b siRNA than in control siRNA-transfected cells (Fig. 1B, lane 3 *vs*. 4).

Further analysis in Panc1 cells revealed that the increase in p-p38 levels in control siRNA-transfected cells after 2 h of TGF-β1 treatment was blunted by ectopic expression of a kinase-dead ALK5 mutant (ALK5_KR_) (Fig. 1C). ALK5_KR_ also prevented the TGF-β1-induced upregulation of p-p38 in Rac1b-depleted cells (Fig. 1C, lane 8 *vs*. 10) and suppressed p-p38 levels in non-TGF-β1-treated control cells (Fig. 1C, compare lane 1 with lane 7 and lane 2 with lane 8). The latter effect may reflect disruption of an autocrine TGF-β signalling loop (see Discussion).

Since the ALK5 kinase phosphorylates both Smads and p38 and phosphorylation of p38 in some cell types has been shown to be Smad-dependent, we sought to reveal whether in Panc1 cells the Rac1b effect on TGF-β1-induced p38 activation is Smad-dependent. To clarify this issue, we employed Panc1 cells stably expressing a dominant-negative mutant of Smad4 (Smad4_1-514_)^26^. We have demonstrated previously that these cells have defective Smad signaling as they failed to respond to TGF-β1 treatment with upregulation of biglycan (BGN)^26^. When we depleted these cells of Rac1b, we observed higher abundance of p-p38 in non-TGF-β1 stimulated Smad4_1-514_ expressing cells vs the corresponding empty vector control cells (Fig. 1D). However, no difference in the response of Smad4_1-514_ expressing cells compared to the vector control cells was seen after 2 and 4 h of TGF-β1 stimulation although there seems to be a trend towards higher p38 activation in Panc1- Smad4_1-514_ cells at 2 h time point (Fig. 1D).

Panc1 cells have been shown to secrete large amounts of TGF-β1 and stimulate themselves in an autocrine fashion^31^. To assess the relative effects of endogenous TGF-β1 vs. rhTGF-β1 on p38 activation upon Rac1b-depletion, we cotransfected Panc1 cells with siRNAs specific for TGF-β1 and Rac1b and determined the levels of p-p38 as above. As shown in Fig. 1E, disrupting endogenous TGF-β1 secretion (verified by a TGF-β1-specific ELISA, Fig. 1E) and hence the autocrine stimulatory feedback loop strongly reduced p-p38 levels in both rhTGF-β1-treated cells (compare lanes 7 and 8 with lanes 5 and 6, and lanes 11 and 12 with lanes 9 and 10) and in non-rhTGF-β1-treated control cells (compare lanes 3 and 4 with lanes 1 and 2) (Fig. 1E). However, while the increase in p-p38 levels between control and Rac1b siRNA-transfected cells was maintained in TGF-β1 siRNA-transfected cells after the 2 h-treatment with rhTGF-β1 (lanes 7 and 8) it was lost after 4 h of treatment (lanes 11 and 12).

Together, these data confirmed our assumption that Rac1b inhibits both an early (<2 h, in Colo357 and HaCaT cells) and a late (2–12 h, in Panc1 cells) phosphorylation response of p38 to rhTGF-β1 stimulation. Moreover, dominant-negative inhibition of ALK5 but not Smad4 in Panc1 cells blunted the TGF-β1-induced upregulation of p-p38 in both control and Rac1b siRNA-transfected cells. Finally, a large fraction of p-p38 under basal conditions is induced by endogenous TGF-β1 and endogenous and rhTGF-β1 cooperate to maintain p-p38 levels in Rac1b-depleted cells higher than in control cells beyond 2 h of stimulation with rhTGF-β1.

### Rac1b depletion results in an increase in basal and TGF-β1-induced p-ERK1/2 levels

**Figure 2:**
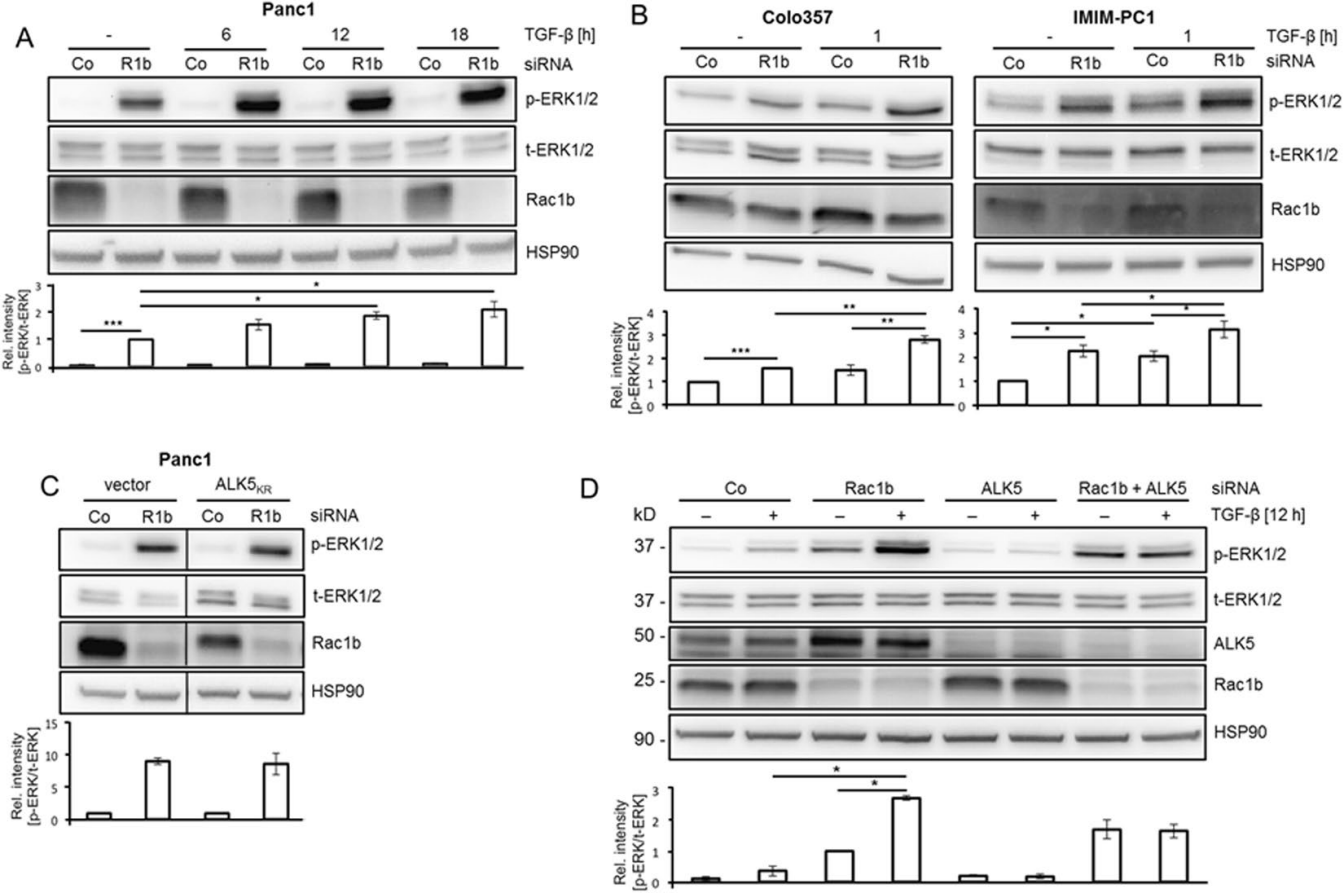
Depletion of Rac1b increases constitutive and growth factor-induced ERK1/2 phosphorylation. (**A**) Panc1 cells were transiently transfected with siRNA specific to Rac1b (R1b) or irrelevant control siRNA (Co), serum-starved for 24 h and treated with TGF-β1 as indicated. Cells were subjected to immunoblotting for p-ERK1/2, t-ERK1/2 and HSP90 as well as Rac1b as a control for transfection efficiency. The chart below shows the relative intensities of the p-ERK1/2 bands normalized to those for t-ERK1/2 from three independent experiments (mean ± SD, n = 3). Data are displayed relative to non-stimulated Rac1b siRNA-transfected cells set arbitrarily at 1. Asterisks indicate a significant difference. (**B**) Colo357 and IMIM-PC1 cells were transiently transfected twice on two consecutive days with 50 nM each of a siRNA specific for Rac1b (R1b), or an irrelevant control siRNA (Co), serum-starved for 24 h and subjected to immunoblotting for phospho-ERK1/2 (p-ERK1/2) and total ERK1/2 (t-ERK1/2) as well al HSP90 as a loading control. The charts below the blots show the relative intensity of the p-ERK1/2 bands normalized to the t-ERK1/2 bands of three independent experiments (mean ± SD, n = 3). Asterisks indicate a significant difference. (**C**) Panc1 cells stably expressing a dominant negative ALK5 mutant (ALK5_KR_) or empty vector were transfected twice with 50 nM each of Co siRNA or Rac1b siRNA and subjected to immunoblot analysis of p-ERK1/2. The blot was stripped and reprobed with antibodies against and t-ERK1/2 and Rac1b. The graph below the blots show the relative intensities of p-ERK1/2 normalized to those for t-ERK1/2 from three independent experiments (mean ± SD, n = 3). (**D**) Panc1 cells were transfected with 50 nM of irrelevant Co siRNA, Rac1b siRNA or ALK5 siRNA, or 25 nM each of Rac1b + ALK5 siRNA. Cells were serum-starved, treated with TGF-β1 for 12 h and subjected to immunoblotting for p-ERK1/2, t-ERK1/2 and HSP90 as well as for Rac1b and ALK5 to verify successful depletion. The graph below the blots shows the relative intensities of p-ERK1/2 bands normalized to those for t-ERK1/2 from three independent experiments (mean ± SD, n = 3). Data are displayed relative to non-stimulated Rac1b siRNA-transfected cells set arbitrarily at 1. The asterisks indicate significance. Data displayed in each panel are from the same blot/gel treated subsequently with antibodies to p-ERK1/2, Rac1b, HSP90, and ALK5, then stripped and reincubated with an antibody to t-ERK1/2.

In PDAC cells ERK1/2 MAPK signalling is activated downstream of Ras and Rac1 but may also be stimulated by TGF-β through the intrinsic tyrosine kinase activity of ALK5^21^. To test whether Rac1b is involved in ERK activation, we monitored by immunoblotting the phosphorylation status of ERK1/2 in various cell lines (Panc1, Colo357, IMIM-PC1 and HaCaT) after siRNA-mediated, selective depletion of endogenous Rac1b. In control siRNA-transfected Panc1 cells we observed an only moderate (2-fold) induction of p-ERK1/2 over a TGF-β1 stimulation period of 18 h (Fig. 2A, lanes 1 *vs*. 7). Rac1b depletion in non-stimulated Panc1 cells enhanced p-ERK1/2 levels by 15-fold (Fig. 2A, lanes 1 *vs*. 2). TGF-β1 stimulation of Rac1b-depleted cells for 12 and 18 h increased ERK levels by 1.9 and 2.1-fold, respectively, relative to non-stimulated control cells (Fig. 2A, lane 2 *vs*. 6, and lane 2 *vs*. 8). In control siRNA-transfected Colo357 and IMIM-PC1 cells p-ERK1/2 was either not induced or induced 2-fold, respectively, within 1 h of TGF-β1 stimulation (Fig. 2B, lane 1 *vs*. 3). Rac1b depletion, however, enhanced p-ERK1/2 levels in non-stimulated Colo357 and IMIM-PC1 cells by 1.6-fold and 2.2-fold, respectively (Fig. 2B, lane 1 *vs*. 2). Importantly, a 1-h treatment with TGF-β1 further enhanced the p-ERK1/2 levels in Rac1b-depleted Colo357 and IMIM-PC1 cells 1.8 and 1.4-fold, respectively, relative to non-stimulated controls (Fig. 2B, lane 2 *vs*. 4). It could be speculated that the changes in p-ERK levels were stronger if a more complete inhibition of Rac1b protein levels would have been obtained in these two cell lines. Very similar results with respect to the effect of Rac1b depletion on TGF-β1 stimulated ERK activation was seen in HaCaT cells (Supplementary Fig. S2).

To test if the Rac1b siRNA-mediated increase in p-ERK1/2 levels in non-stimulated cells was the result of relief from a TGF-β autocrine feedback loop, we blocked TGF-β signalling in Panc1 cells by either ectopic expression of kinase-inactive ALK5_KR_ (Fig. 2C) or by siRNA to ALK5 (Fig. 2D). In Panc1-ALK5_KR_ cells, p-ERK1/2 levels were not different from those in control cells after Rac1b depletion (Fig. 2C). Likewise, in Panc1 cells codepleted of ALK5 and Rac1b protein by siRNA levels of p-ERK1/2 appeared enhanced rather than decreased relative to cells only depleted of Rac1b (Fig. 2D, lane 3 *vs*. 7). However, as expected, ALK5 siRNA prevented appearance of the TGF-β1-induced fracton of p-ERK1/2 protein in Rac1b siRNA-transfected cells (Fig. 2D, lane 4 *vs*. 8).

To study the impact of combined Rac1 + Rac1b depletion on ERK activation, we transfected Panc1 cells with a Rac1 siRNA (which targets both Rac1 and Rac1b). In contrast to selective depletion of Rac1b, codepletion of Rac1 *and* Rac1b did not result in a statistically significant increase in p-ERK1/2 levels (Supplementary Fig. S3), suggesting that while Rac1b inhibition promotes ERK1/2 activation under basal conditions, concomitant inhibition of Rac1 has the opposite effect and was able to override the Rac1b siRNA effect.

### Ectopic overexpression of Rac1b decreases TGF-β-dependent ERK1/2 activation and inhibits TGF-β target gene expression

**Figure 3:**
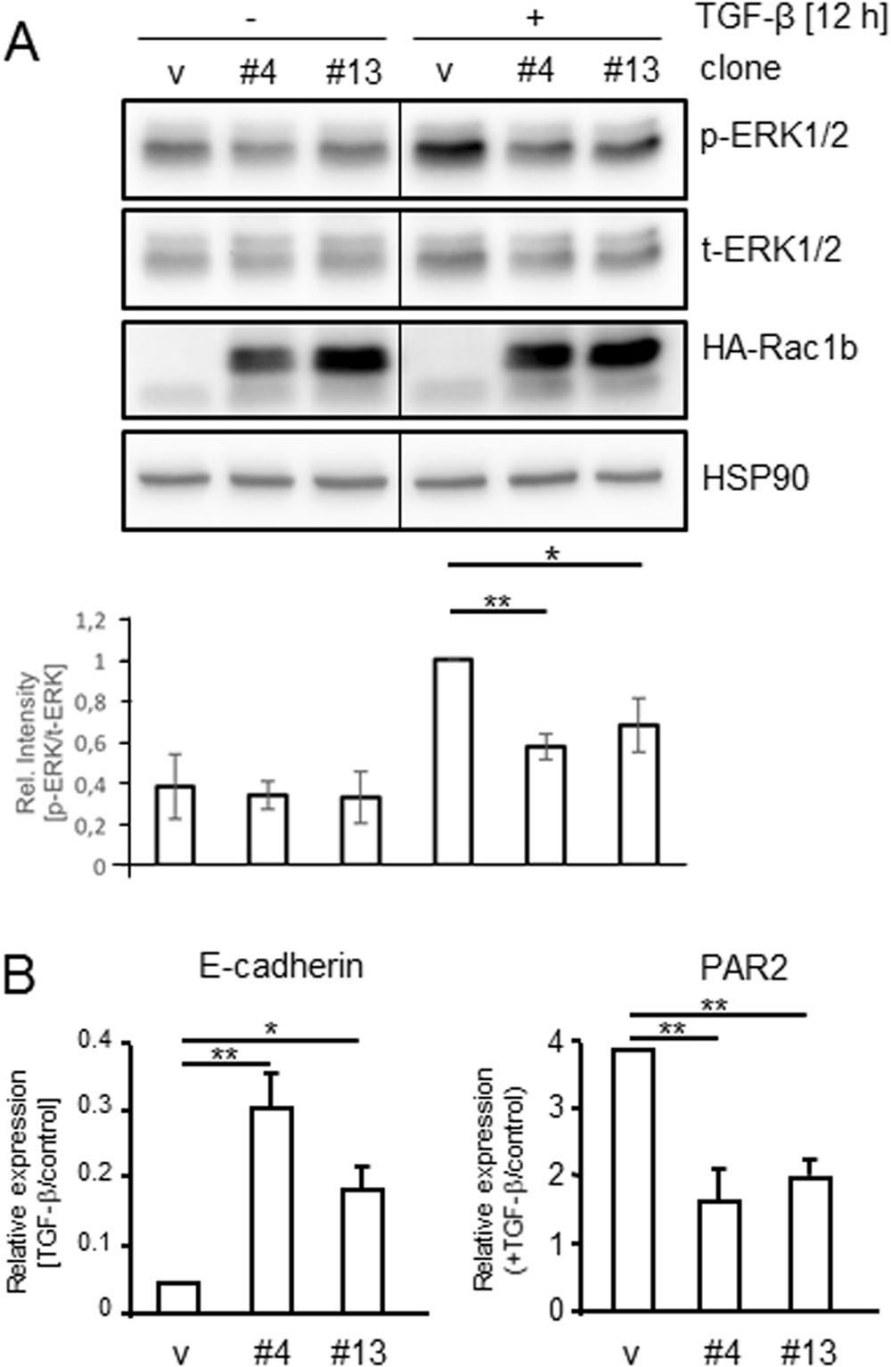
Effects of ectopic overexpression of Rac1b on TGF-β1-induced ERK1/2 activation and gene expression. (**A**) Panc1 cells stably overexpressing Rac1b from the pCGN vector (two independent clones, #4 and #13) as well as empty-vector control cells (v) were serum-starved, treated with TGF-β1 for 12 h and subjected to immunoblotting for p-ERK1/2, t-ERK1/2, and HA-Rac1b. The graph underneath the blot shows results from densitometric analysis of the respective bands for p-ERK1/2 normalized to those for t-ERK1/2 and derived from four independent experiments (mean ± SD, n = 4). Data are displayed relative to TGF-β1 stimulated vector control cells set arbitrarily at 1. Asterisks indicate a significant difference of p-ERK1/2 band intensity between the vector control and the two HA-Rac1b expressing clones. Due to the short exposure time, endogenous Rac1b protein is not visible. (**B**) The same cells as in (A) were stimulated with TGF-β1 for 24 h and subjected to qPCR for E-cadherin and PAR2. Data are displayed as TGF-β1-treated over control cells and are the mean ± SD of 3 experiments. Asterisks indicate significant differences. Data are from the same blot/gel treated subsequently with antibodies to p-ERK1/2, Rac1b, and HSP90, then stripped and reincubated with an antibody to t-ERK1/2. The vertical lines between lanes 3 and 4 indicate removal of irrelevant lanes from the blot.

In a previous study, we have shown that stable ectopic overexpression of HA-Rac1b in PDAC cells suppresses TGF-β1-mediated cell migration^9^. To study if this effect is associated with a concomitant downregulation of signalling pathways promoting cell motility such as Ras-MEK-ERK1/2, we monitored p-ERK1/2 levels in two previously characterised independent HA-Rac1b expressing clones of the Panc1 cell line^9^ after 12 h of TGF-β1 treatment (the time of maximal ERK activation, see Fig. 2A). To this end, p-ERK1/2 levels were significantly reduced in both clones relative to empty vector controls clones (Fig. 3A).

To analyse whether ectopic Rac1b also impacts regulation of other TGF-β target genes we studied in the same cells regulation of E-cadherin, an EMT-associated gene that is downregulated by this growth factor, and regulation of proteinase-activated receptor 2 (PAR2), a G protein-coupled receptor implicated in tumour cell invasion/metastasis^32^ and required for a full-blown TGF-β response^28^. In line with previous results on TGF-β-dependent expression of Slug^9^, downregulation of E-cadherin and upregulation of PAR2 by TGF-β1 was greatly reduced in both clones (Fig. 3B). Together with the data presented in Fig. 2, this indicates that Rac1b is a negative regulator of TGF-β1-induced ERK activation and gene expression in PDAC-derived cells.

### RNAi-mediated depletion of Rac1b enhances the expression of genes associated with TGF-β1-induced EMT

**Figure 4:**
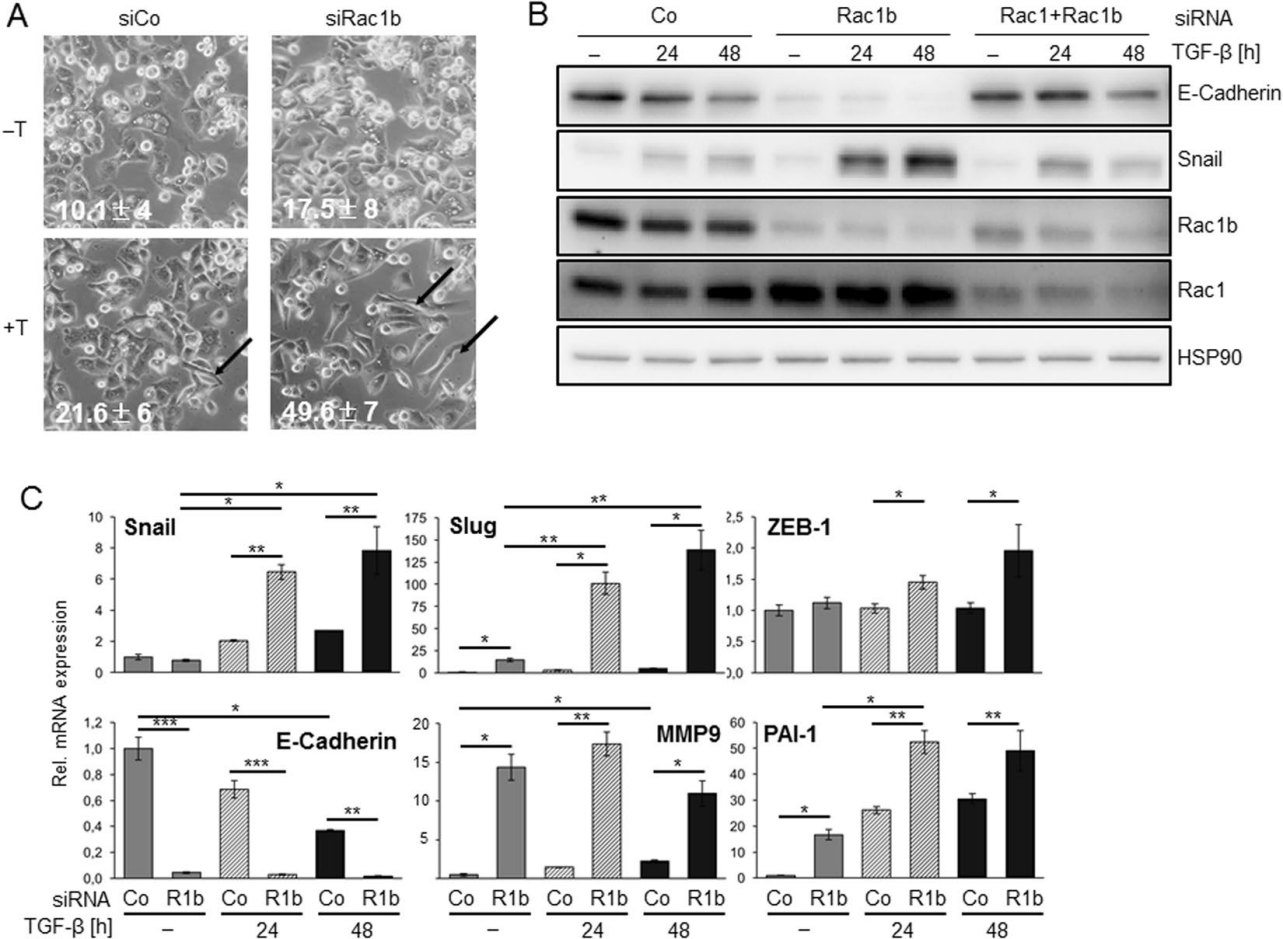
Effect of Rac1b depletion on EMT-associated genes. (**A**) Phase-contrast images of Panc1 cells transfected with an irrelevant control siRNA (siCo) or siRNA to Rac1b (siRac1b) and subsequently treated for 48 h or not (-) with 5 ng/ml TGF-β1 in medium with 0.5% FBS. Three experiments were performed in total, of which one is shown. The arrows point to cells with an elongated morphology. Insets indicate the percentage of spindle-shaped cells per visual field (mean ± SD, n = 3, independently counted by two investigators), p = 0.0039 for siCo + T *vs*. siRac1b + T and p = 0.0025 for siRac1b-T *vs*. siRac1b + T. Magnification: ×400. (**B**) Panc1 cells were transfected with an irrelevant control siRNA (Co), siRNA specific to Rac1b, or siRNA targetting both Rac1 and Rac1b. Transfected cells were treated with TGF-β1 for 24 or 48 h in medium containing 0.5% FBS and then subjected to sequential immunoblotting for E-Cadherin and Snail, Rac1 and Rac1b to verify functionality of the siRNAs, and HSP90 as loading control without intermittent stripping. The bands shown are all from the same blot/gel. Three experiments were performed in total of which one is shown. **c** Panc1 cells were transfected with Rac1b (R1b) or control (Co) siRNA, serum-starved and treated with TGF-β1 for 24 and 48 h. Cells were then subjected to qPCR-based expression analysis for the indicated genes. Slug was used as positive control^9^. Successful Rac1b depletion (21.4 ± 0.0069% of Co) was verified by qPCR using exon 3b-specific primers. Data are given as Relative (Rel.) mRNA expression and represent the mean ± SD from at least three independent experiments. Asterisks indicate significant differences.

Cell migration is known to be associated with the process of EMT which involves distinct changes in cellular morphology and gene expression. Having shown that depletion of Rac1b enhanced TGF-β1-induced cell migration while its ectopic overexpression partially prevented downregulation of E-cadherin (see Fig. 3B), we considered the possibility that Rac1b depletion also enhances EMT induced by TGF-β1. To study this in more detail, we again depleted Panc1 cells of cellular Rac1b protein by RNAi and subsequently exposed the cells for 48 h to TGF-β1. Interestingly, Rac1b depletion enhanced the ability of TGF-β1 to induce cell scattering and the appearance of cells with a spindle-shaped morphology (Fig. 4A). To analyse if these changes correspond to alterations in cell adhesion molecules or transcription factors orchestrating the EMT process, we performed immunoblot analysis for E-cadherin and Snail, two proteins that are down- and upregulated, respectively, during TGF-β-induced EMT. Strikingly, E-cadherin and Snail protein levels in control siRNA-transfected cells decreased and increased, respectively, by TGF-β1 treatment (Fig. 4B). Upon Rac1b depletion E-cadherin levels were dramatically reduced independent of TGF-β while those of Snail were strongly increased only in TGF-β1-treated cells (Fig. 4B, lanes 4–6). Interestingly, cells could be rescued from the Rac1b effect when transfected with an siRNA directed against both Rac1b and Rac1 (Fig. 4B, lanes 7–9). We then employed qPCR analysis to clarify whether Rac1b regulation of E-Cadherin and Snail as well as other EMT/migration-associated TGF-β target genes was evident at the transcriptional level. To this end, Rac1b regulation of E-cadherin and Snail mRNA essentially mirrored the immunoblot data (Fig. 4C). A similar regulatory pattern as for Snail mRNA was also seen for Slug and ZEB-1 mRNA. Rac1b depletion sensitised Panc1 cells to Slug and ZEB-1 mRNA induction by TGF-β1 that was higher and lower, respectively, than for Snail (Fig. 4C). Likewise, mRNA of matrix metalloproteinase 9 (MMP9) and plasminogen activator-inhibitor type I (PAI-1), two proteins involved in TGF-β-induced invasion of PDAC cells^33^, were both markedly upregulated after Rac1b knockdown while PAI-1 but not MMP9 mRNA levels in Rac1b-depleted cells were further enhanced by concomitant stimulation with rhTGF-β1 for 24 h (Fig. 4C).

### Inhibition of p38 and ERK1/2 MAPK signalling prevents TGF-β1 dependent hyperexpression of EMT marker expression after Rac1b silencing

**Figure 5:**
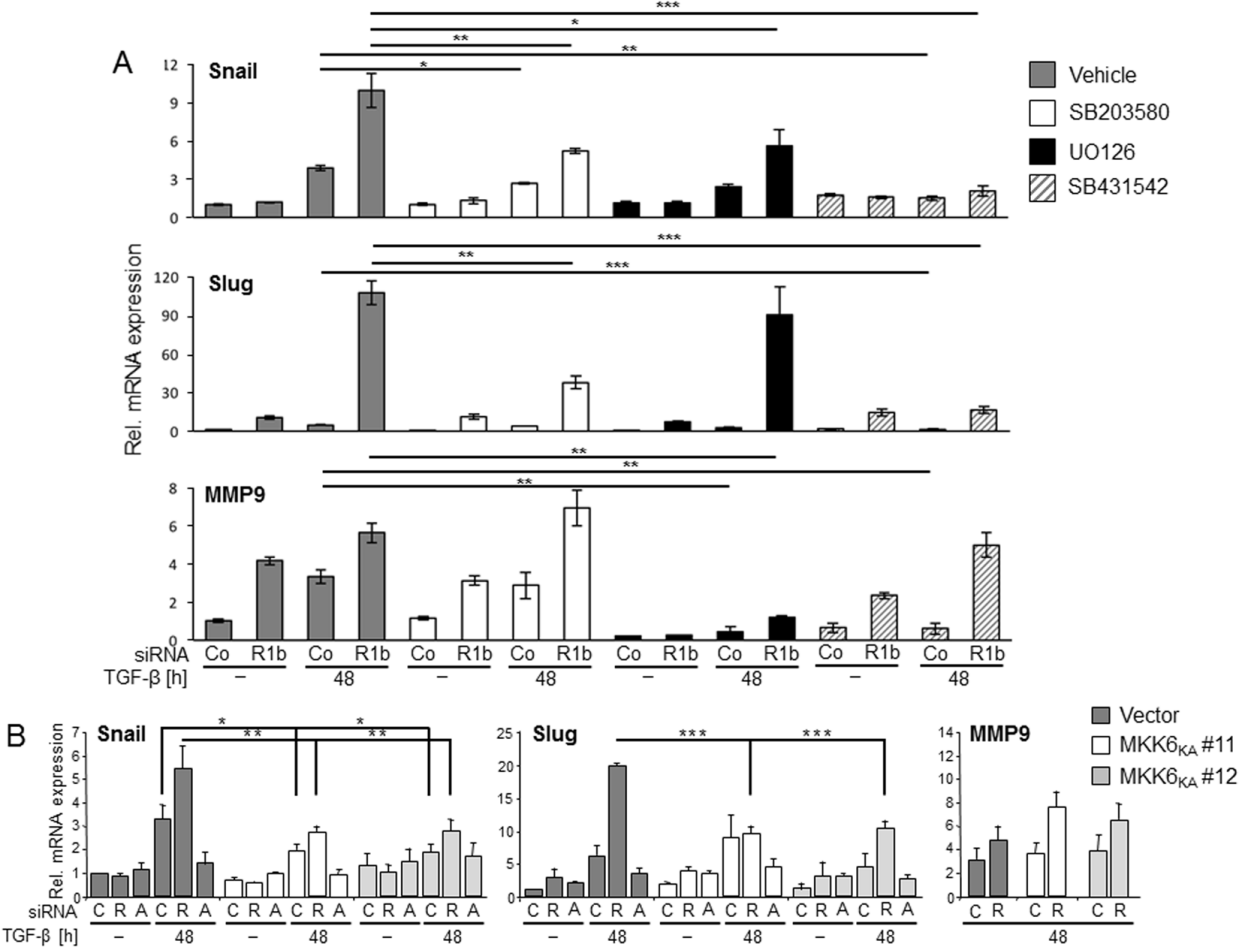
Inhibition of p38 and ERK1/2 MAPKs reverses the EMT-associated changes in gene expression after depletion of Rac1b. Panc1 cells were transfected twice with siRNA specific to Rac1b or control siRNA, serum-starved overnight and treated with SB203580 (10 μM), UO126 (10 μM), SB431542 (5 μM) or vehicle (DMSO, 0.1%) as a control. Thirty min after addition of inhibitors, cells received TGF-β1 treatment and were incubated for 48 h in medium with 0.5% FBS followed by qPCR analysis. Pilot studies indicated that cell viability was not impaired by treatment with either of these inhibitors for 48 h (not shown). (**A**) Expression of Snail, Slug, and MMP9 mRNA in Rac1b-depleted and inhibitor-treated Panc1 cells. Successful Rac1b depletion was verified by qPCR as in Fig. 4C (not shown). Three experiments were performed with similar results, of which one is shown. Asterisks indicate a significant decrease in gene expression in TGF-β1 + inhibitor-treated, Rac1b-depleted cells compared to the respective vehicle-treated cells (mean ± SD, n = 3). (**B**) Effect of transfection of various siRNAs (C, control, R, Rac1b; A, ALK5) on Snail, Slug, and MMP9 expression in two individual clones of Panc1 cells (#11, #12) ectopically expressing a dominant-negative mutant of MKK6 (MKK6_KA_) from a retroviral vector or empty vector expressing control cells. The transfection and TGF-β1 treatment procedure was performed as described in (**A**). For MMP9, only the data from TGF-β1-treated and control and Rac1b siRNA-transfected cells are shown. Three experiments were performed and data shown are the mean ± SD. Asterisks indicate a significant decrease in gene expression in Rac1b-depleted and TGF-β1-treated MKK6_KA_ *vs*. empty vector cells.

As shown above, Rac1b knockdown sensitised the Snail, Slug and PAI-1 genes in Panc1 cells to hyperinduction by TGF-β1 which may in part reflect a relief from Rac1b inhibition of Smad2/3 C phosphorylation^9^. However, TGF-β1-mediated control of EMT and cell motility in PDAC cells does not only require Smad, but also crosstalk with p38 MAPK - and in pancreatic cancer cells with activating K-Ras mutations - with the Ras-Raf-MEK-ERK-signalling cascade. We therefore analysed if the dramatic increase in gene expression of Snail, Slug, PAI-1, and MMP-9 after Rac1b removal was sensitive to inhibition of p38 and/or ERK1/2 MAPK signalling. Panc1 cells depleted of Rac1b were treated or not with TGF-β1 in the presence or absence of the specific p38 MAPK inhibitor SB203580 (10 μM), the MEK inhibitor U0126 (10 μM), or the ALK5 inhibitor SB431542 (5 μM) as control. Results show that in Rac1b-depleted cells both SB203580, U0126, and SB431542 as control, were able to reduce the TGF-β1 effect on Snail mRNA (Fig. 5A) and Snail protein (Supplementary Fig. S4) expression, while the stimulatory effect of Rac1b depletion on TGF-β1-induced Slug was only sensitive to SB203580 and SB431542 (Fig. 5A). In contrast, TGF-β1/Rac1b siRNA-mediated hyperstimulation of PAI-1 was not affected by SB203580 but was sensitive to U0126 and SB431542 (data not shown). TGF-β-ALK5 signalling to *MMP9* has been reported to be dependent on MEK-ERK but not JNK, p38 or Smad4^34^. Interestingly, MMP9 mRNA upregulation after Rac1b siRNA transfection was completely prevented by U0126 in both control and Rac1b-depleted cells, while SB203580 and SB431542 had no major effect (Fig. 5A).

We then strived to confirm the results from pharmacologic inhibition by ectopic expression of MKK6_KA_, a dominant negative mutant of the p38 upstream activator MKK6. In agreement with the data from pharmacological inhibition, Rac1b siRNA + TGF-β1-induced hyperexpression of Snail and Slug was strongly reduced in both MKK6_KA_ clones compared to empty vector expressing control cells. In contrast and in agreement with the small molecule inhibition data (see Fig. 5A), Rac1b siRNA + TGF-β1-induced upregulation of PAI-1 mRNA (not shown) and MMP9 mRNA (Fig. 5B) in MKK6_KA_ clones did not differ significantly from vector control cells. As expected, the stimulatory effect of TGF-β1 on Snail and Slug expression in both MKK6_KA_ and empty vector expressing Panc1 cells was blunted upon transfection with an ALK5 siRNA (Fig. 5B).

In summary, hyperinduction of TGF-β target gene expression by Rac1b depletion in combination with TGF-β1 treatment was differentially relieved by various inhibitors reflecting involvement of the specific non-Smad/MAPK pathways involved. While hyperinduction of Snail mRNA was blocked by inhibition of either MKK6-p38 or MEK-ERK signalling, Rac1b siRNA-mediated overexpression of Slug mRNA was only blocked by inhibition of MKK6-p38 signalling and that of PAI-1 and MMP9 mRNA only by inhibition of MEK-ERK signalling.

### Inhibition of p38 or ERK1/2 MAPK signalling abolishes the promigratory effect of Rac1b silencing

**Figure 6:**
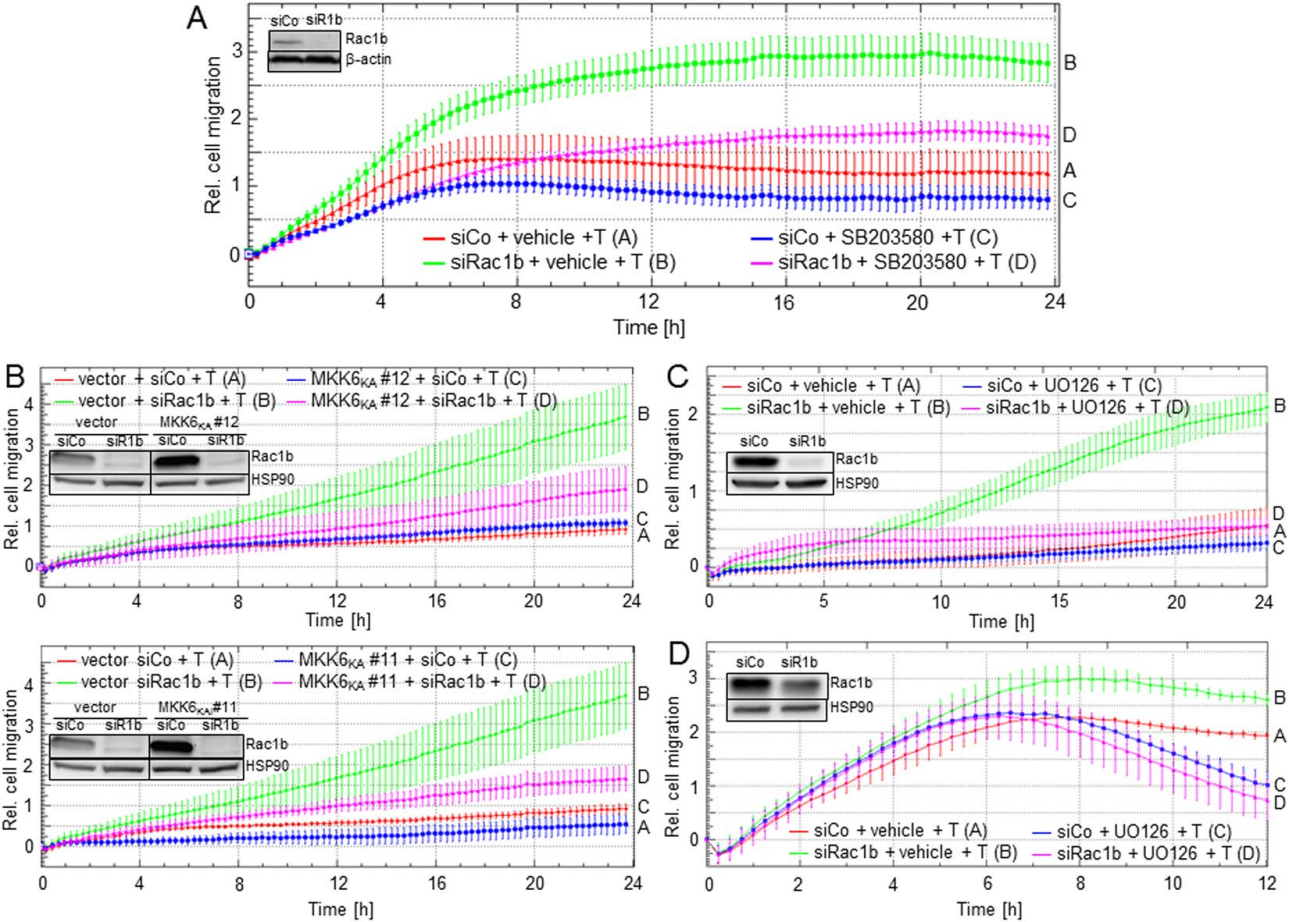
Inhibition of the p38 and ERK MAPK pathways alleviates the stimulatory effect of Rac1b knockdown on TGF-β1-induced migration. (**A**) Panc1 cells were transfected with siRNA specific for Rac1b (siRac1b) or an appropriate control siRNA (siCo). Transfected cells were serum-starved overnight and subjected to xCELLigence cell migration assay, in the presence of TGF-β1 (T, 5 ng/ml) and either SB203580 (10 µM) or vehicle (DMSO, 0.1%). Cell migration was measured every 15 min for 24 h. Letters to the right of each graph allow for a colour-independent identification of the various curves. Differences between siRac1b-transfected vehicle (green curve/tracing B) and SB203580 (magenta curve/tracing D) treated cells become significant as early as 2 h and remain so at all later time points. (**B**) Individual clones of Panc1 cells expressing MKK6_KA_ or empty-vector control cells were transiently transfected with siRac1b or siCo. Transfected cells were serum-starved and subjected to xCELLigence cell migration assay in presence of 5 ng/ml TGF-β1. Cell migration was recorded every 15 min for 24 h and the results for two individual clones plotted against the control cells are shown. Differences between siRac1b-transfected vector cells (green curves/tracings B) and MKK6_KA_ expressing cells (magenta curves/tracings D) from both clones become significant at the 22 h time point. (**C**) Panc1 cells transiently transfected with siRac1b or siCo and subsequently treated with TGF-β1 were analysed by real-time cell migration assay in the absence or presence of U0126 (10 µM). The difference between siRac1b-transfected vehicle-treated cells (green curve/tracing B) and U0126-treated cells (magenta curve/tracing D) is significant at the 10 h and all later time points. (**D**) As in (**C**), except that IMIM-PC1 cells were used. The difference between siRac1b-transfected cells treated with vehicle (green curve/tracing B) and those treated with U0126 (magenta curve/tracing D) is significant at the 8 h and all later time points. In each panel, a representative experiment is shown from three experiments performed in total with similar results. Note that for the sake of clarity, only the data from TGF-β1-treated cells are shown.

Cell migration and invasion are considered EMT-associated processes that are controlled by both Smad and non-Smad, *e.g*. MAPK signalling. We therefore hypothesised that Rac1b exerts its antimigratory/antiinvasive effects via inhibition of either the MKK6-p38 and/or the MEK-ERK pathway. When Rac1b-depleted cells were subjected to xCELLigence® technology-based real-time cell migration assays, the promigratory effect of TGF-β1 was dramatically enhanced (Fig. 6A, green *vs*. red curve). Notably, treatment of cells with SB203580 during the assay reduced the TGF-β1-induced migration of the Rac1b siRNA-transfected cells at the 24 h time point by 59 ± 20.5% (n = 3, p < 0.05) compared to siRac1b-transfected cells treated with vehicle (Fig. 6A, green *vs*. magenta curve). We then repeated the migration assays in MKK6_KA_ expressing Panc1 cells. The relative migratory activity of TGF-β1-treated Rac1b siRNA-transfected MKK6_KA_ expressing clones #11 and #12 at the 24 h time point was reduced by 60 ± 8% (n = 3, p < 0.05) and 46 ± 28% (n = 3, p < 0.05), respectively, compared to Rac1b siRNA-transfected empty vector expressing control cells (Fig. 6B, green *vs*. magenta curve. Note that for the sake of clarity only the curves for TGF-β1-treated cells are shown).

The MAPKs ERK1/2 are also crucial in TGF-β-dependent EMT and cell motility of PDAC-derived cells^18^. Interestingly, the MEK inhibitor U0126 completely blocked the pro-migratory TGF-β1 effect in Rac1b-depleted cells (Fig. 6C, note that only the curves for TGF-β1-treated cells are shown). In IMIM-PC1 cells, Rac1b depletion also enhanced the TGF-β1 effect on random cell migration only in vehicle treated cells (Fig. 6D, green *vs*. magenta curve, significant at 4 h and all later time points) but not in U0126-treated cells (Fig. 6D, red *vs*. blue curve, no significant difference at any time point). Together, these data indicate that Rac1b depletion-induced enhancement of the promigratory TGF-β1 effect in PDAC cells could be blocked by inhibition of either p38 or ERK1/2 signalling.

### Rac1b depletion enhances TGF-β1-induced growth arrest

**Figure 7:**
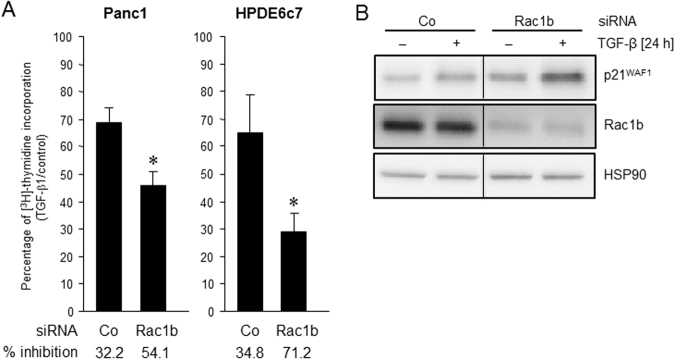
SiRNA-mediated knockdown of Rac1b enhances TGF-β1-mediated growth inhibition. (**A**) Panc1 or HPDE6c7 cells were transiently transfected twice with 50 nM each of Co siRNA or Rac1b siRNA followed by another 24-h incubation in normal growth medium. Cells were then stimulated, or not, with TGF-β1 for 24 h. In every transfection, aliquots of cells were tested for successful inhibition of siRNA targets by qPCR analysis before and after the assay (not shown). DNA synthesis in [^3^H]-thymidine-pulsed cells was measured during the last 4 h of the TGF-β stimulation period. Data represent the mean ± SD of six wells processed in parallel. Asterisks indicate significance relative to the respective Co siRNA-transfected sample. Data are displayed as % inhibition of [^3^H]-thymidine uptake after TGF-β1 treatment relative to untreated control cells set arbitrarily to 100%. The assays were performed three times and data represent the mean ± SD (n = 3). (**B**) Panc1 cells were transfected with Rac1b siRNA and treated with TGF-β1 as outlined under **(A)**, and subjected to sequential immunoblotting for p21^WAF1^, Rac1b and HSP90 without intermittent stripping. The bands shown are all from the same blot/gel and the vertical lines indicate removal of irrelevant lanes. Three experiments were performed of which one is shown.

Data so far indicate that Rac1b through inhibiting p38 and ERK activation suppresses EMT-associated changes in morphology, gene expression, and cell motility. Another prominent response to TGF-β, growth inhibition, has also been implicated in TGF-β-induced EMT and according to our hypothesis should also be under negative control by Rac1b. Preliminary evidence for this came from the microscopy and cell counting experiments in which it was observed that cultures treated with a combination of Rac1b siRNA and TGF-β1 appeared more sparse (see Fig. 4A). To this end, siRNA-mediated depletion of Rac1b resulted a much stronger growth inhibitory effect of TGF-β1 in both Panc1 cells and in the non-tumourigenic pancreatic ductal epithelial cell line HPDE6c7 as measured by DNA synthesis via [^3^H]-thymidine incorporation (Fig. 7A). Interestingly, the greater ability of TGF-β1 to inhibit DNA synthesis in Rac1b-depleted Panc1 cells correlated with elevated protein levels of the cyclin-dependent kinase inhibitor p21^WAF1^ (Fig. 7B). Together, these data show that Rac1b acts as an inhibitor of TGF-β1-induced growth inhibition.

### Rac1b depletion alters the expression of positive and negative regulators of TGF-β signalling towards an inhibitory outcome

**Figure 8:**
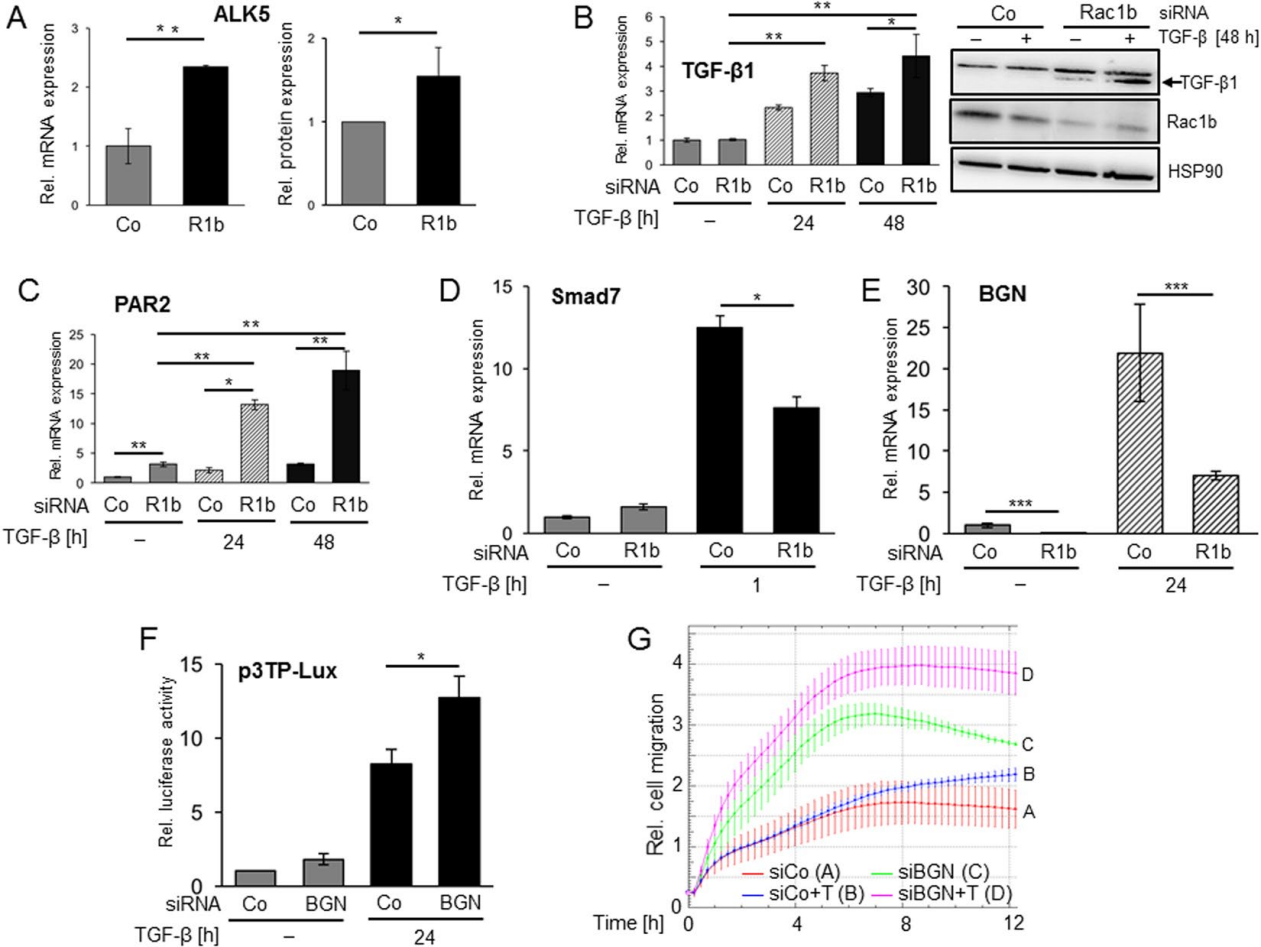
Effects of Rac1b depletion on the expression of positive and negative regulators of TGF-β signalling. (**A**) Panc1 cells were transiently transfected twice with 50 nM each of Rac1b (R1b) siRNA or control (Co) siRNA. Forty-eight h after the second transfection cells were subjected to either qPCR (left) or immunoblot (right) analysis for ALK5 expression. Immunoblot data were quantified by densitometry and represent the mean ± SD of 6 experiments. Asterisks indicate significant differences. (**B**) Panc1 cells were transiently transfected twice with 50 nM each of R1b siRNA or Co siRNA, serum-starved and treated with TGF-β1 for the indicated times. Cells were then subjected to either qPCR (left) or immunoblot (right) analysis for TGF-β1 expression. The immunoblot (all bands are from the same blot/gel) was sequentially treated without intermittent stripping with antibodies to TGF-β1, Rac1b, and HSP90. (**C**) As in (**B**), except that cells were subjected to qPCR analysis of PAR2 expression. (**D**) As in (**B**), except that cells were treated for only 1 h with TGF-β1 and subjected to qPCR analysis of Smad7 expression. (**E**) As in (**B**), except that cells were subjected to qPCR analysis of BGN expression. In (**A–E**) three experiments were performed each and data represent the mean ± SD of three assays (n = 3). Asterisks indicate significant differences. (**F**) Panc1 cells were transiently transfected with 50 nM each of Co siRNA or BGN siRNA along with the TGF-β-responsive luciferase reporter plasmid p3TP-Lux. Twenty-four h later, cells were subjected to dual luciferase assay. Luciferase data (mean ± SD) were derived from 6 wells processed in parallel and corrected for transfection efficiency with *Renilla* luciferase activity measured in the same sample. Shown is a representative assay out of five assays performed in total. (**G**) Panc1 cells were transiently transfected twice with 50 nM each of siRNA specific for BGN (siBGN) or a scrambled control siRNA (siCo). Transfected cells were serum-starved overnight and subjected to xCELLigence cell migration assay in absence or presence of TGF-β1 (T, 5 ng/ml). Two experiments with very similar results were conducted of which one is shown. Differences in the migratory activity between TGF-β1-treated siCo-transfected cells (blue curve/tracing B) and siBGN-transfected cells (magenta curve/tracing D) become significant at the 1 h time point, and those between their non-stimulated counterparts (red curve/tracing A and green curve/tracing C, respectively) at the 3 h time point.

Data so far indicate that Rac1b is a potent inhibitor of TGF-β/ALK5-induced EMT, expression of EMT/migration-associated genes as well as activation of Smad2/3^9^ and p38 and ERK MAPKs (this study). Rac1b might thus control the TGF-β pathway in a more direct and subtle fashion, *i.e*. by regulating the expression of the TGF-β receptor(s) or TGF-β ligand(s). Strikingly, we consistently observed an upregulation of ALK5 protein abundance following silencing of Rac1b (Fig. 1C: ectopically expressed ALK5_KR_, lanes 7–12, and Fig. 2D: endogenous ALK5, lanes 1 and 2 vs. lanes 3 and 4). A more thorough and quantitative analysis at both the RNA and protein levels confirmed this observation (Fig. 8A). Moreover, TGF-β1 exhibits autoinduction of its expression (Fig. 8B) and, likewise, the TGF-β1-dependent levels of TGF-β1 mRNA (Fig. 8A, graph) and protein (Fig. 8B, immunoblot) were further enhanced upon Rac1b depletion. Of interest, PAR2 was dramatically increased when Rac1b depletion was combined with TGF-β1 exposure (Fig. 8C) and these data were in line with impaired TGF-β1 induction of PAR2 mRNA in HA-Rac1b overexpressing cells (see Fig. 3B).

Above, we observed a greatly prolonged period of p38 activation in response to TGF-β1 stimulation in Rac1b-depleted cells which might be explained by defective termination of TGF-β signalling. We therefore tested whether Rac1b also affects the expression of negative regulators of TGF-β signalling such as the inhibitory Smad, Smad7. Smad7 terminates excess signalling activity of activated ALK5 by preventing the binding of Smad2/3 and by enhancing internalization and ubiquitin-mediated degradation of ALK5^35^. Intriguingly, Rac1b depletion resulted in a *decrease* in Smad7 expression in TGF-β1-treated cells (Fig. 8D), suggesting that Rac1b, in addition to inhibiting initiation of TGF-β signalling, also favors its termination through Smad7 upregulation.

Activation of the TGF-β signalling pathway is primarily determined by the abundance, bioavailability, and access of the TGF-β ligand to the receptors. Hence, cells may protect themselves from overstimulation by soluble TGF-β by secreting factors such as BGN that can bind and sequester TGF-β, prevent its binding to TβRII/ALK5 and thus neutralise its biological activity^36^. Given this function, we hypothesised that i) BGN is *positively* controlled by Rac1b and if so ii) down-regulation of BGN should be able to mimic the Rac1b siRNA effect and amplify TGF-β1-induced reporter gene activity and cell migration. To test this hypothesis, we chose Panc1 cells because BGN expression is dramatically induced by TGF-β1 in these cells within a period of 24 h^26^. Strikingly, and as shown above for Smad7, BGN induction was strongly *reduced* after Rac1b silencing in both untreated control cells and in cells treated for 24 h with TGF-β1 (Fig. 8E). Moreover, silencing *BGN* with a specific siRNA was associated with enhanced TGF-β/Smad transcriptional activity of the TGF-β-dependent luciferase reporter gene p3TP-Lux (Fig. 8F). Moreover, in both untreated and TGF-β1-treated Panc1 cells BGN depletion caused a marked *increase* in their migratory activity (Fig. 8G). Together, these findings suggest that Rac1b interferes with TGF-β pathway activation in several ways, *e.g*. by inhibiting the expression of positive regulators (ALK5, TGF-β1, PAR2) and by promoting the expression of negative ones such as Smad7 and BGN.

## Discussion

We have shown previously that Rac1b interfered with TGF-β1-dependent activation of Smad2/3 and random cell migration^9^. Since TGF-β induction of cell motility in PDAC-derived cells involves Smad and non-Smad signalling pathways, which may be activated independently from each other, it was of interest to reveal whether TGF-β1-induced p38 and/or ERK signalling, too, are subject to (negative) control by Rac1b. Notably, Rac1b depletion in Panc1 cells in a Smad4-independent manner prevented the decline in p-p38 levels seen at 4 h of TGF-β1 stimulation and extended the duration of the p38 phosphorylation response to TGF-β1 to 12 h. Of note, this time course correlated closely with the kinetics of the migratory response of this cell line to TGF-β1 (see Fig. 6A,B). A similar albeit less pronounced effect of Rac1b depletion was seen in Colo357 cells after 4 and 8 h of TGF-β stimulation but not earlier which may be due to less effective inhibition of Rac1b protein (due to a generally lower transfection efficiency) in this cell line when compared to Panc1 cells (see Supplementary Fig. S1). In HaCaT cells, Rac1b depletion had no effect on basal p-p38 levels but potentiated the TGF-β1-induced p-p38 levels at the 1 h time point (see Fig. 1B).

Activation of ERK1/2 by TGF-β1 in PDAC cells is somewhat variable and can comprise an early and/or a late activation episode depending on the cell type^18^. However, Rac1b depletion led to a strong increase in basal p-ERK1/2 levels and this was further enhanced by rhTGF-β1 treatment (see Fig. 2A,B). In contrast to Panc1 cells, a rapid activation (within 1 h) is seen in both control and Rac1b siRNA-transfected Colo357 and IMIM-PC1 cells (see Fig. 2B, compare lanes 1 and 3), and in HaCaT cells (see Supplementary Fig. S2). The strong ERK activation upon RNAi-mediated downregulation of Rac1b protein remained unaltered in Panc1 cells ectopically expressing ALK5_K232R_ (see Fig. 2C), transfected with ALK5 siRNA (Fig. 2D), or treated with the ALK5 inhibitor SB431542 (not shown), suggesting that the Rac1b siRNA-induced upregulation of p-ERK1/2 levels is independent of the TGF-β/ALK5 pathway. In contrast, the additional increase in ERK1/2 phosphorylation elicited by rhTGF-β1 in Rac1b-depleted cells was blunted by ALK5 protein depletion (see Fig. 2D). In addition, we analysed the effects of ectopic overexpression of HA-Rac1b in individual clones of Panc1 cells. In two independent clones we observed a *reduced* ability of TGF-β1 to induce ERK1/2 activation relative to empty vector controls (see Fig. 3). Hence, our data so far point to a crucial role of Rac1b in negative control of TGF-β1-dependent activation of p38 and ERK in PDAC and non-PDAC cells. The ability of Rac1b to effectively suppress non-Smad TGF-β signalling may underlie its potent anti-EMT and antimigratory activity. Interestingly, a negative effect of Rac1b on MEK-ERK signalling has also been observed upon neurotrophin 3 (NT3) stimulation of human bone marrow-derived stromal cells^37^.

A major issue of this study was whether selectively inhibiting the generation of Rac1b protein would affect the cells’ response to TGF-β1-induced EMT. Notably, Rac1b depletion enhanced the ability of TGF-β1 to induce morphological changes such as a spindle-shaped morphology and altered the expression of various epithelial and mesenchymal EMT marker genes. Specifically, Rac1b depletion alone (without addition of rhTGF-β1) dramatically enhanced downregulation of E-cadherin and upregulation of Slug, MMP9, and PAI-1 expression. Moreover, cells depleted of Rac1b protein reacted with a much stronger induction by rhTGF-β1 of Snail, Slug, ZEB-1, and PAI-1 mRNA. For E-cadherin and Snail we confirmed this regulatory pattern at the protein level (Fig. 4B). Taken together with the inhibitory effect of Rac1b on TGF-β1-dependent Smad2/3 activation^9^, the data implicate Rac1b as a protein that helps to maintain an epithelial phenotype thereby protecting PDAC-derived cells from TGF-β1-induced mesenchymal conversion.

Using pharmacological and dominant-negative inhibition strategies, we show that blocking p38 signalling partially relieved the Rac1b siRNA-induced hypererinduction by TGF-β1 for Snail and Slug, but not PAI-1 and MMP9 (see Fig. 5). Blocking Ras-Raf-MEK-ERK signalling with U0126 completely or partially relieved the Rac1b siRNA-induced hypererinduction by TGF-β1 for Snail, PAI-1, and MMP9, respectively, but not Slug. With respect to the signalling pathways involved, regulation of MMP9 deserves particular attention since TGF-β-ALK5 signalling to *MMP9* is dependent solely on MEK-ERK but not JNK, p38 or Smad4^34^. This has been confirmed here by the dramatic upregulation of MMP9 mRNA after Rac1b siRNA transfection (Figs 4C and 5A) and its almost complete relief by U0126 in both control and Rac1b-depleted Panc1 cells (Fig. 5A) and by the failure of MKK6_KA_ expression (Fig. 5B), SB203580 and SB431542 (Fig. 5A) to mimic the U0126 effect. The failure of SB431542 to decrease the MMP9 mRNA levels in Rac1b-depleted and TGF-β1-treated cells is likely due to the fact that ERK activation by TGF-β is mediated by the tyrosine kinase rather than the serine/threonine kinase function of ALK5^21^. Our data on p38 involvement in TGF-β regulation of E-cadherin, Snail, and PAI-1 are in agreement with published data^38,39^. Moreover, we revealed differences in TGF-β-MAPK signalling to Snail and Slug in PDAC cells. Our data show that by inhibiting p38 and ERK signalling, Rac1b can suppress the response of critical regulators of TGF-β-induced EMT program. To confirm the important role of non-Smad-mediated TGF-β signalling in Rac1b regulation of EMT, we additionally evaluated the effects of the above mentioned p38 and ERK inhibitors on TGF-β1-stimulated cell migration. In agreement with the gene expression data, the strong increase in TGF-β1-induced migratory activity resulting from Rac1b depletion could be relieved by treatment of cells with SB203580 or U0126, or by ectopic expression of a dominant-negative mutant of MKK6.

EMT program has been associated with enhanced growth inhibition^40^ which contributes to the known chemo- and radioresistant phenotype of PDAC cells. Interestingly, Rac1b appears to act as an inhibitor of TGF-β1-dependent growth arrest in malignant and benign pancreatic ductal epithelial cells (see Fig. 7A), probably by suppressing the expression of p21^WAF1^, a potent cell cycle inhibitor in PDAC cells^41^. This matches the role of Rac1b as an EMT inhibitor and suggests the exciting possibility that Rac1b can be utilized as a chemo-/radiosensitzer in targeted therapies for PDAC. In this context it is noteworthy that inhibition of TGF-β signalling has been discussed as a potential strategy to improve success of radiotherapy^42^ and of novel therapies based on the use of TRAIL^43^.

Prompted by the negative effects of Rac1b on EMT-associated gene expression, migration, and growth inhibition as well as on Smad and MAPK signaling, we pursued the idea that Rac1b targets central components of the TGF-β signalling pathway. Intriguingly, Rac1b appears to restrict expression of TGF-β1 ligand, ALK5, and PAR2, a G protein-coupled receptor that was shown recently by us to be indispensable for Smad activation and TGF-β1-dependent cell motility^28^. While the TGF-β1, ALK5 and PAR2 genes are all under negative control by Rac1b, two endogenous inhibitors of TGF-β signalling, namely Smad7 and BGN, turned out to be positively regulated by Rac1b reinforcing the concept of Rac1b being an effective inhibitor of TGF-β signalling. The observation that ALK5 protein was more abundant in Rac1-depleted cells is particularly interesting since altering receptor expression is a prominent mechanism through which tumour cells can modulate their sensitivity to TGF-β^44^.

PDAC cells, particularly Panc1 and to a lower extent Colo357 and IMIM-PC1 cells, are known to secrete large amounts of TGF-β1 into the culture medium and to autostimulate themselves^31^. Since in Panc1 cells exposed to rhTGF-β1 Rac1b controls TGF-β1 mRNA and protein expression in a negative fashion, its depletion is expected to enhance this autocrine loop by relieving TGF-β-Smad and MAPK signalling from inhibition. The presence of an autocrine feedback loop is also suggested by the observation that the p-p38 levels in both non-rhTGF-β1 stimulated control siRNA and Rac1b siRNA-transfected cells were reduced upon dominant-negative inhibition of the ALK5 kinase (see Fig. 1C) and siRNA-mediated depletion of endogenous TGF-β1 (see Fig. 1E).

With respect to its possible role in tumour progression, our data suggest that Rac1b is required for maintaining an epithelial phenotype by preventing TGF-β1 from inducing EMT and thus a mesenchymal and potentially invasive phenotype in the tumour cells. These results provide a molecular correlate for the antimetastatic function proposed earlier by us on the basis of higher expression of Rac1b in long-time *vs*. short-time survivors among PDAC patients^9^.

Crosstalk of TGF-β with K-Ras signalling has been shown to be central to tumourigenesis of PDAC^1^, however, therapeutically targetting K-Ras may not be feasible as available Ras inhibitors have largely failed to block the protumourigenic effects of oncogenic K-Ras. Since Rac1 is a well-known downstream target of Ras and mediator of TGF-β1-induced EMT and cell motility, our observation that Rac1b can antagonise Rac1 function and help to maintain an epithelial phenotype in PDAC-derived cells is intriguing. Therapeutically increasing the generation of Rac1b over that of Rac1 in the tumour tissue, *e.g*. by shifting the splice ratio^45^ could be a means to block some undesired effects of hyperactive Ras and may represent a promising strategy for alleviating the protumourigenic effects of TGF-β.

## Electronic supplementary material

Supplementary figures and tables
