# Supplementary material for: Negative regulation of TGF-β1-induced MKK6-p38 and MEK-ERK signalling and epithelial-mesenchymal transition by Rac1b
Source: Sci Rep. 2017 Dec 11;7:17313. doi: 10.1038/s41598-017-15170-6 (PMC5725500; doi:10.1038/s41598-017-15170-6)

Supplementary Figures and Tables

Title: Negative regulation of TGF-β1-induced MKK6-p38 and MEK-ERK signalling and epithelial-mesenchymal transition by Rac1b

Authors: David Witte, Hannah Otterbein, Maria Förster, Klaudia Giehl, Robert Zeiser, Hendrik Lehnert, Hendrik Ungefroren

Supplementary Table S1: Primers used for qPCR

**Primer name**	**Sequence (5'→3')**	**GenBank accession**
ALK5-forward	GCGACGGCGTTACAGTGTTTCTGC	NM_004612
ALK5-reverse	ATGGTGAATGACAGTGCGGTTGTGG	NM_004612
β-actin-forward	GACGAGGCCCAGAGCAAGAG	NM_001101
β-actin-reverse	ATCTCCTTCTGCATCCTGTC	NM_001101
Biglycan-forward	CCCTCTCCAGGTCCATCCGC	NM_001711
Biglycan-reverse	GAGCTGGGTAGGTTGGGCGGG	NM_001711
E-cadherin-forward	TCTTCCCCGCCCTGCCAATC	Z13009
E-cadherin-reverse	GCCTCTCTCGAGTCCCCTAG	Z13009
GAPDH-forward	TTGCCATCAATGACCCCTTCA	NM_001289745
GAPDH-reverse	CGCCCCACTTGATTTTGGA	NM_001289745
MMP9-forward	CATTTCGACGATGACGAGTTGT	NM_004994
MMP9-reverse	CGGGTGTAGAGTCTCTCGC	NM_004994
PAI-1-forward	CTTCTTCAGGCTGTTCCGGAGC	X04744
PAI-1-reverse	GGGTCAGGGTTCCATCACTTGG	X04744
Rac1b-forward	GGAGAAACGTACGGTAAGGATATAACC	NM_018890
Rac1b-reverse	GGCAATCGGCTTGTCTTTGCCC	NM_018890
Smad7-forward	GGAAGATCAACCCCGAGCTG	NM_005904
Smad7-reverse	TTGGGAATCTGAAAGCCCCC	NM_005904
Snail-forward	CTGCTCCACAAGCACCAAGAGTC	NM_005985
Snail-reverse	CCAGCTGCCCTCCCTCCAC	NM_005985
Slug-forward	ATATTCGGACCCACACATTACCT	NM_003068
Slug-reverse	GCAAATGCTCTGTTGCAGTGA	NM_003068
PAR2-forward	ACTCCAGGAAGAAGGCAAACA	NM_005242
PAR2-reverse	TGGTCTGCTTCACGACATACA	NM_005242
TGF-β1-forward	CCCATGCCGCCCTCCGGGCTGC	NM_000660
TGF-β1-reverse	TCAGCTGCACTTGCAGGAGC	NM_000660
TBP-forward	GCTGGCCCATAGTGATCTTT	M55654.1
TBP-reverse	CTTCACACGCCAAGAAACAG	M55654.1
ZEB-1-forward	TCCATGCTTAAGAGCGCTAGCT	NM_001128128
ZEB-1-reverse	ACCGTAGTTGAGTAGGTGTATGCCA	NM_001128128

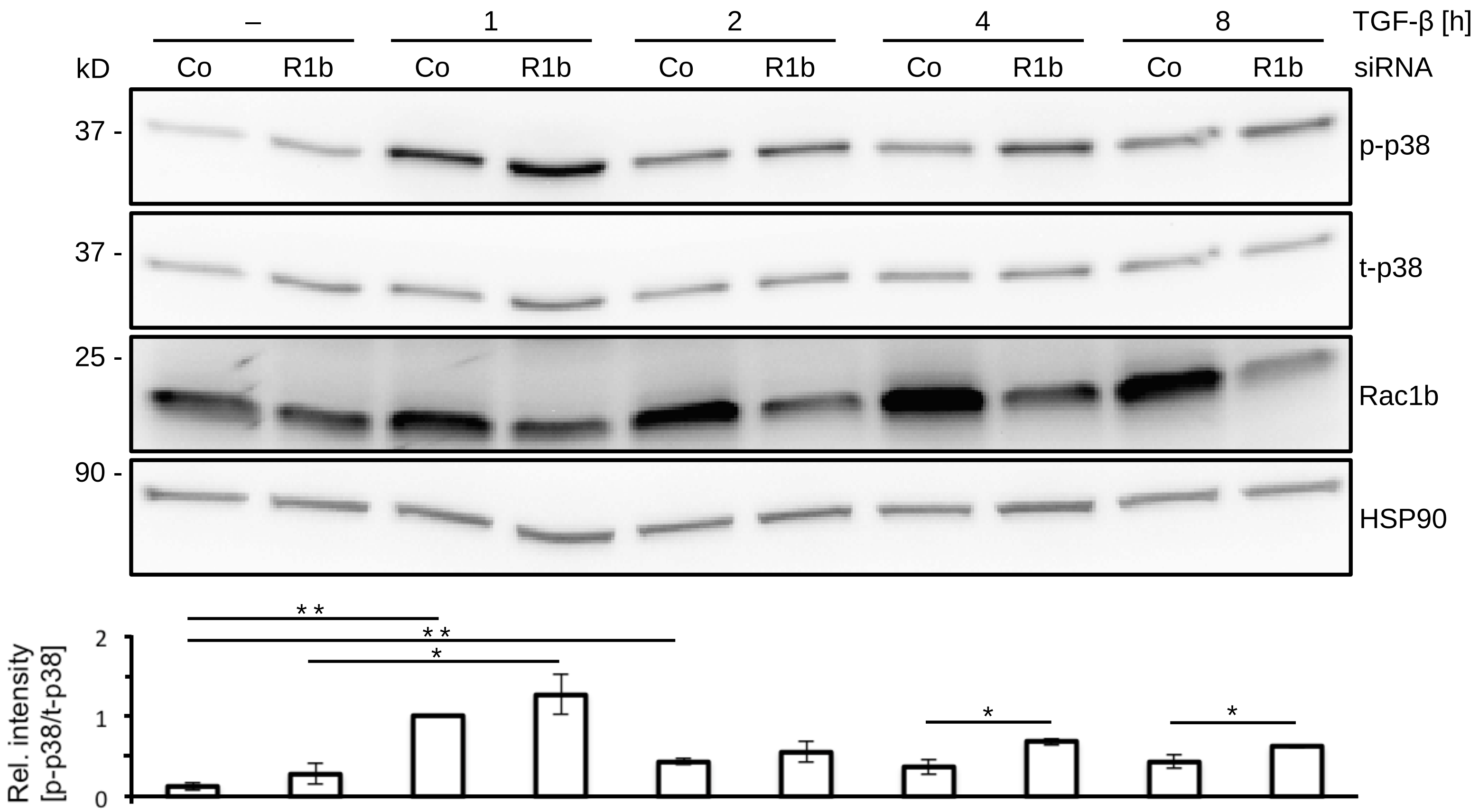

Supplementary Figure S1. Effect of inhibition of Rac1b expression in the PDAC-derived cell line Colo357. Colo357 cells were transfected with siRNA specific for Rac1b (R1b) or control siRNA (Co), serum-starved for 24 h and treated with 5 ng/ml TGF-β1 as indicated. Cells were then subjected to immunoblotting for p-p38 MAPK, t-p38 MAPK and HSP90 as controls for equal loading as well as Rac1b as a control for transfection efficiency. The chart below the blots shows the relative band intensities for p-p38 normalized to those for t-p38 from three independent experiments (mean ± SD). Data are displayed relative to control siRNA transfected cells treated for 1 h with TGF-β1 (set arbitrarily at 1). Asterisks indicate significant differences.

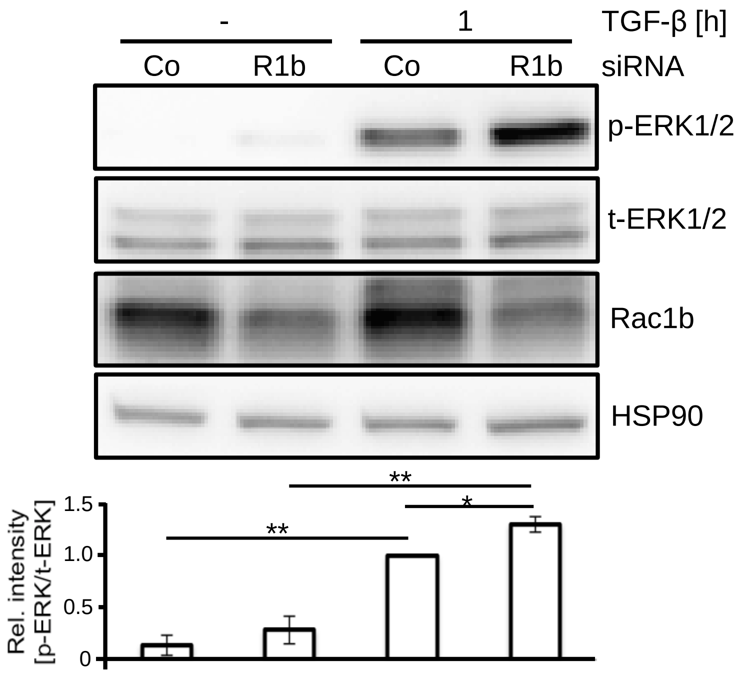

Supplementary Figure S2. Cellular depletion of Rac1b in keratinocytes enhances the TGF-β effect on ERK activation. HaCaT cells were transfected with siRNA specific for Rac1b (R1b) or control siRNA (Co), serum-starved for 24 h and treated with 5 ng/ml TGF-β1 for 1 h. Cells were then subjected to immunoblotting for p-ERK1/2, t-ERK1/2, and HSP90 for verification of equal loading and Rac1b for control of transfection efficiency. The chart below the blots shows the mean relative intensity of the p-ERK1/2 bands normalized to t-ERK1/2 and represent the mean ± SD of three experiments. Data are displayed relative to control siRNA transfected and TGF-β1 treated cells set arbitrarily at 1. Asterisks indicate significant differences.

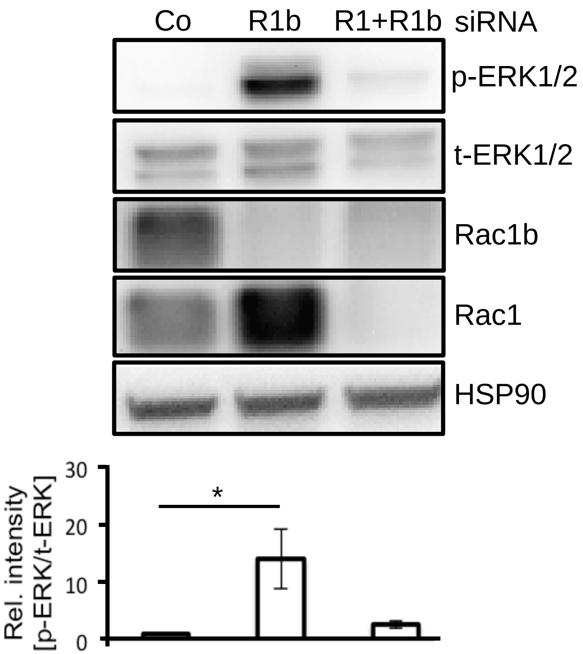

Supplementary Figure S3. Codepletion of Rac1 and Rac1b does not increase ERK activation. Panc1 cells were transfected with irrelevant control siRNA (Co), siRNA specific to Rac1b (R1b), or siRNA to Rac1 *and* Rac1b (R1+R1b). Cells were serum-starved for 24 h and subjected to immunoblotting for p-ERK1/2, t-ERK1/2 as well as Rac1b and Rac1 to verify successful depletion. The chart below shows the relative band intensities for p-ERK1/2 normalized to those for t-ERK1/2 from three independent experiments (mean ± SD, n=3). The asterisk indicates a significant difference.

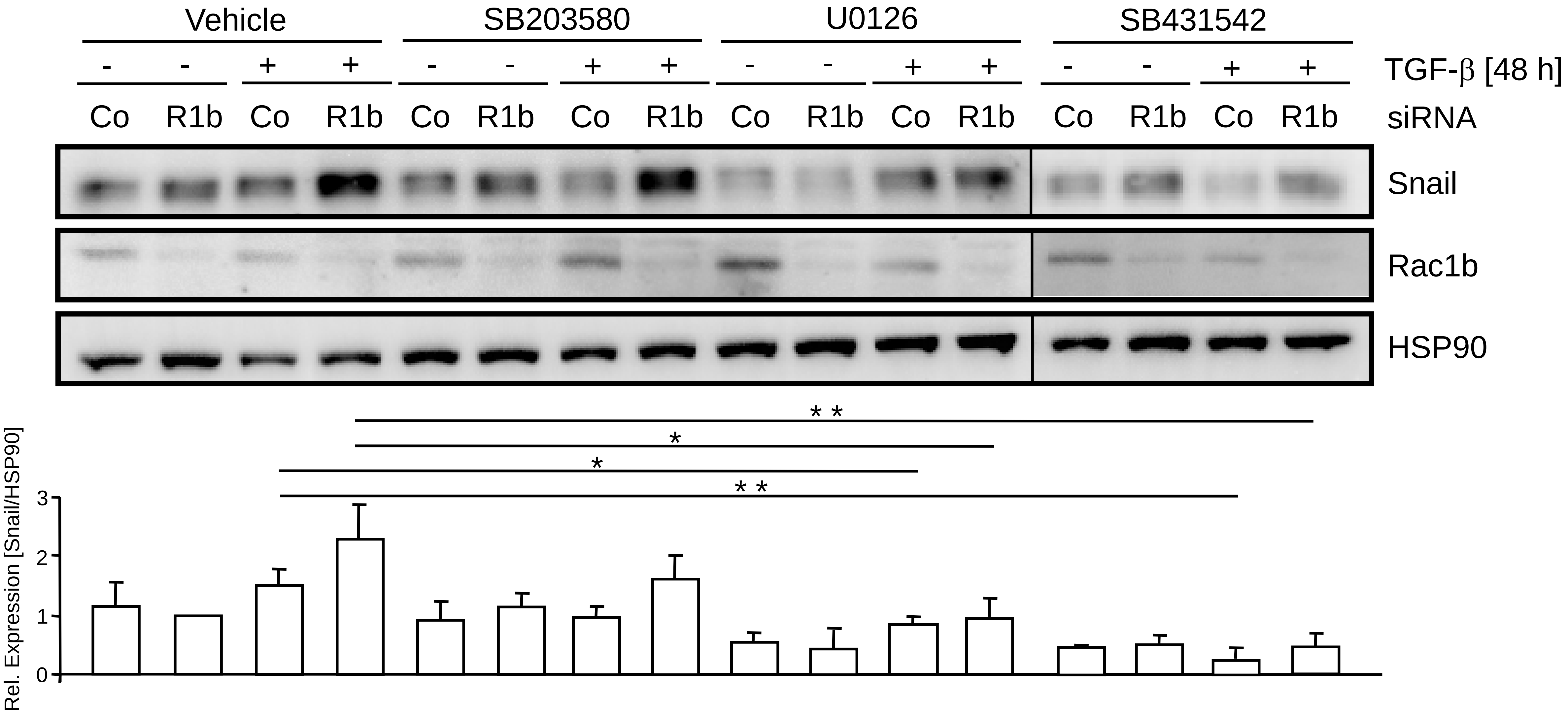

Supplementary Figure S4. Effect of inhibition of p38 and ERK1/2 MAPK signalling on Rac1b siRNA-induced Snail expression. Panc1 cells were transfected twice with siRNA specific to Rac1b (R1b) or control (Co) siRNA, serum-starved overnight and treated with SB203580 (10 µM), UO126 (10 µM), SB431542 (5 µM) or vehicle (DMSO, 0.1%) as a control. Thirty min after addition of inhibitors, cells received TGF-β1 treatment and were incubated for 48 h in medium with 0.5% FBS. Cells were then subjected to immunoblotting for Snail, Rac1b (as a control for transfection efficiency), and HSP90 (as a control for equal loading). The chart below the blots shows the relative band intensities for Snail normalized to those for HSP90 from three independent experiments (mean ± SD). Data are displayed relative to Co siRNA transfected, non-TGF-β1 but DMSO-treated cells (set arbitrarily at 1). Asterisks indicate significant differences. The vertical lines between lanes 12 and 13 indicates removal of irrelevant lanes from the blot.

Supplementary Information file: Full-length gels and blots

Title: Negative regulation of TGF-β1-induced MKK6-p38 and MEK-ERK signalling and epithelial-mesenchymal transition by Rac1b

Authors: David Witte, Hannah Otterbein, Maria Förster, Klaudia Giehl, Robert Zeiser, Hendrik Lehnert, Hendrik Ungefroren

Figure 1A

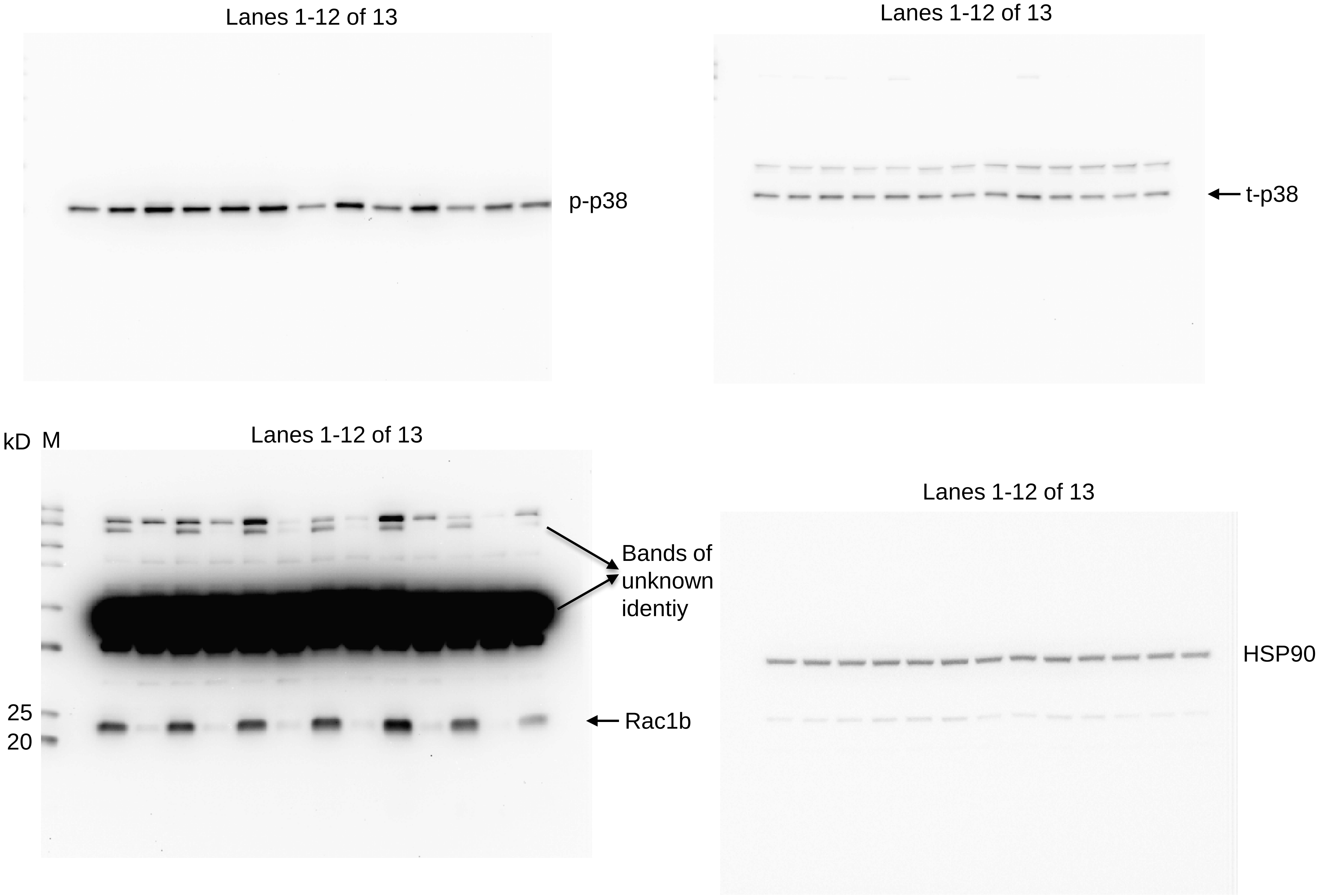

M = molecular weight marker (Precision Plus Protein WesternC Standards, BioRad, Catalog #161-0376)

Figure 1B

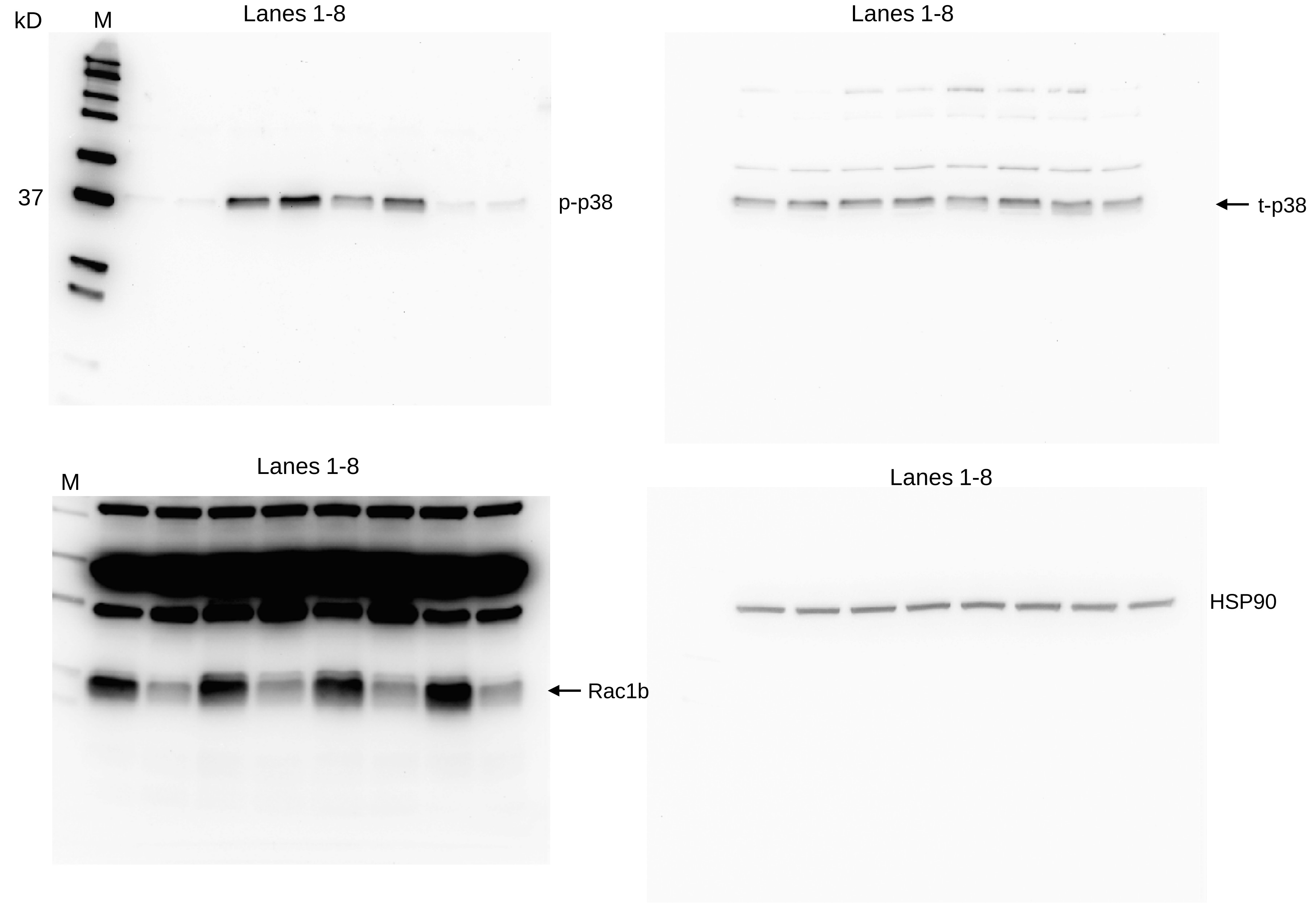
kD
M
Lanes 1-8
37
p-p38
Lanes 1-8
t-p38
M
Lanes 1-8
Rac1b
Lanes 1-8
HSP90

Figure 1C

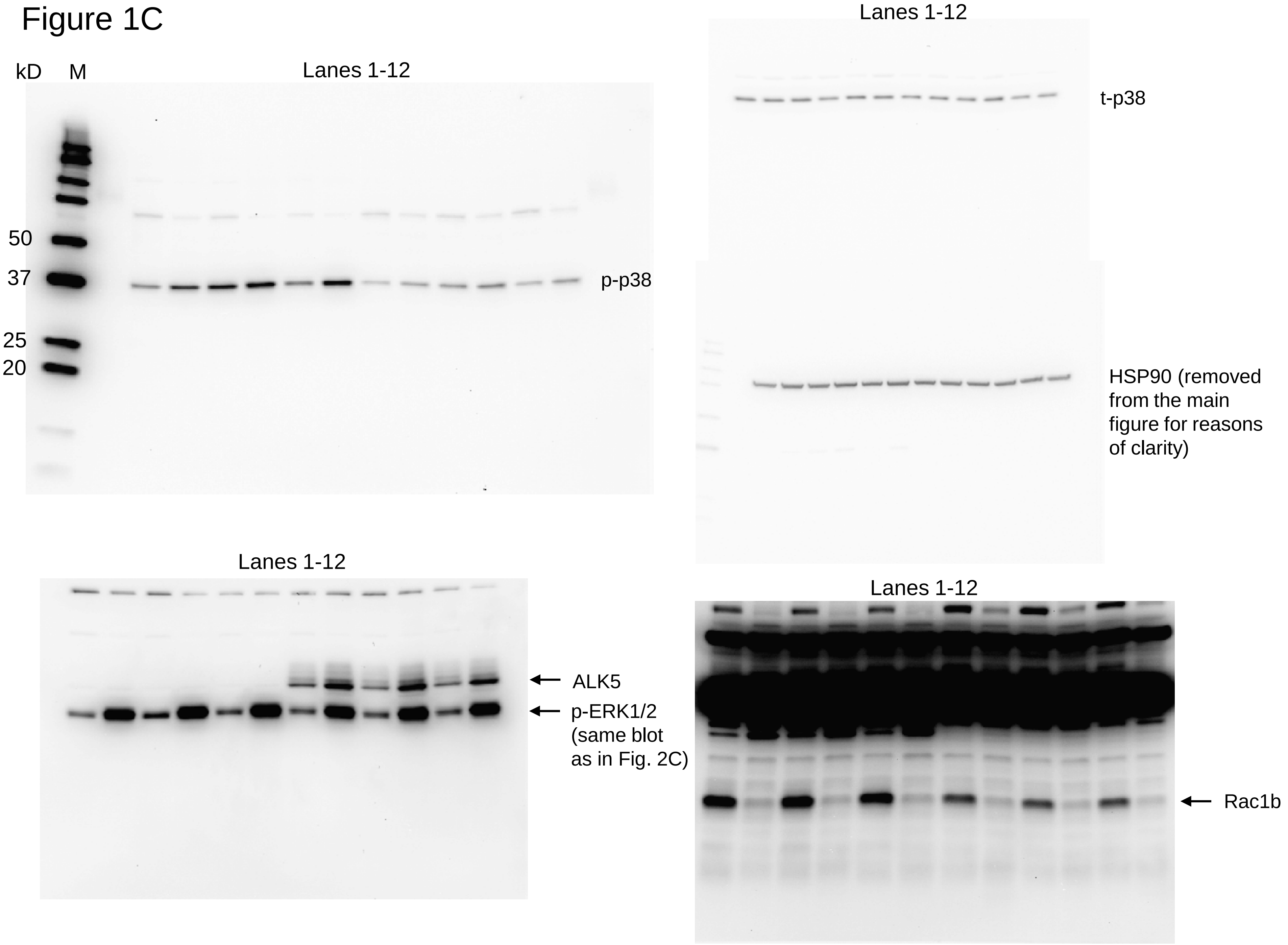
kD
M
Lanes 1-12
50
37
25
20
p-p38
Lanes 1-12
t-p38
HSP90 (removed from the main figure for reasons of clarity)
Lanes 1-12
ALK5
p-ERK1/2 (same blot as in Fig. 2C)
Lanes 1-12
Rac1b

Figure 1D

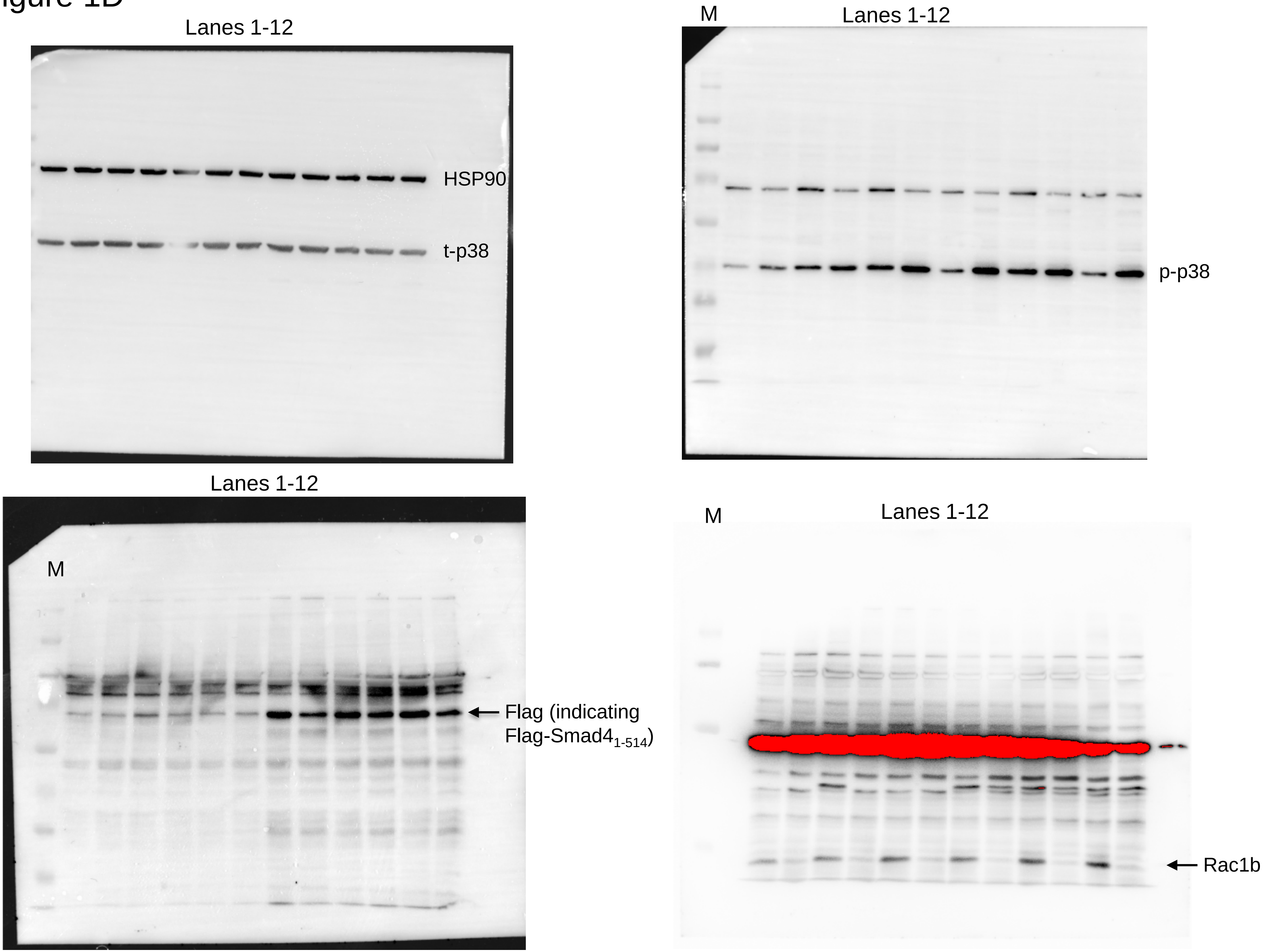

Lanes 1-12
HSP90
t-p38
M
Lanes 1-12
p-p38
Lanes 1-12
M
Flag (indicating
Flag-Smad4$_{1-514}$)
M
Lanes 1-12
Rac1b

Figure 1E

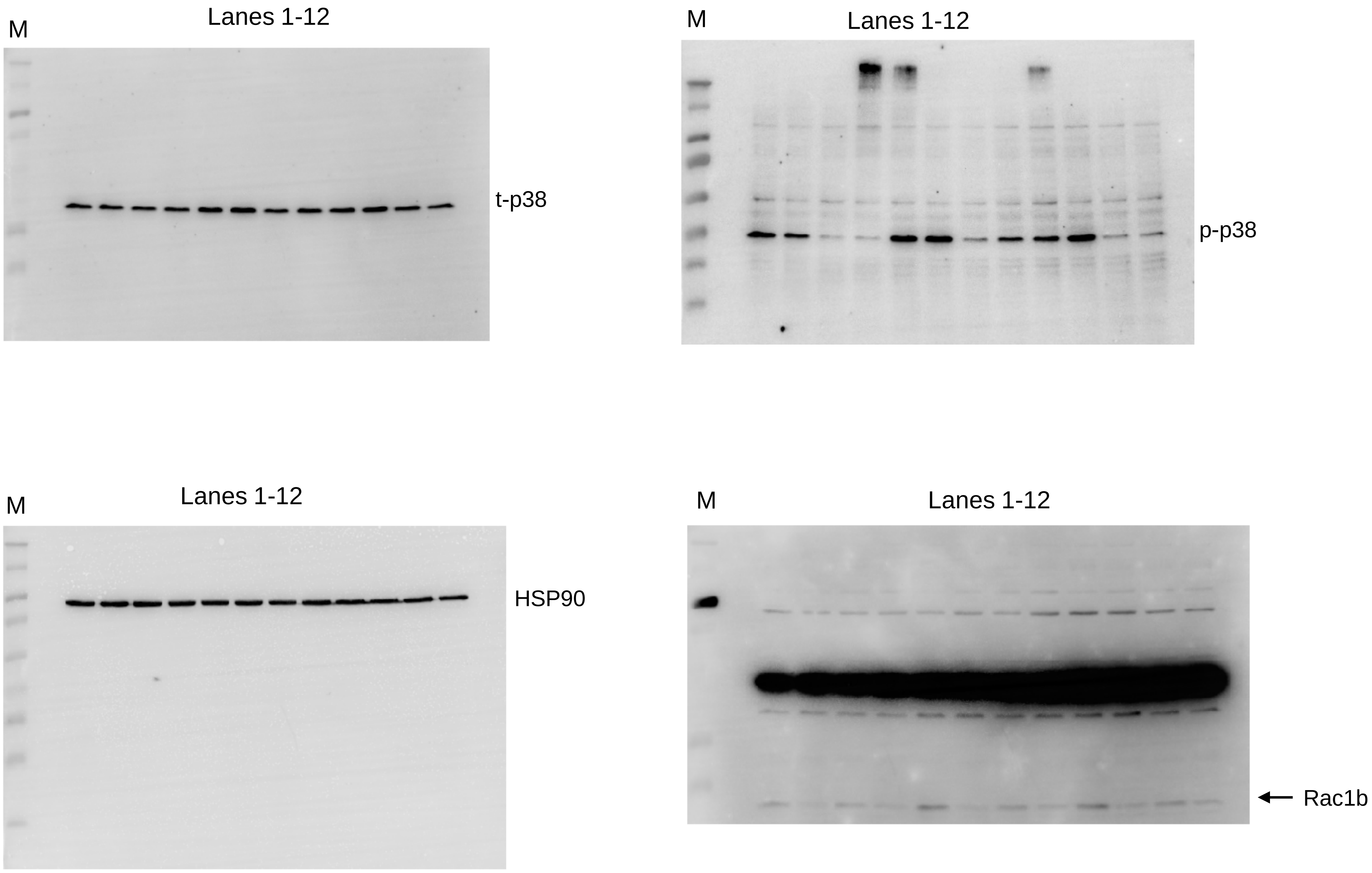
M
Lanes 1-12
t-p38
M
Lanes 1-12
p-p38
M
Lanes 1-12
HSP90
M
Lanes 1-12
Rac1b

Figure 2A

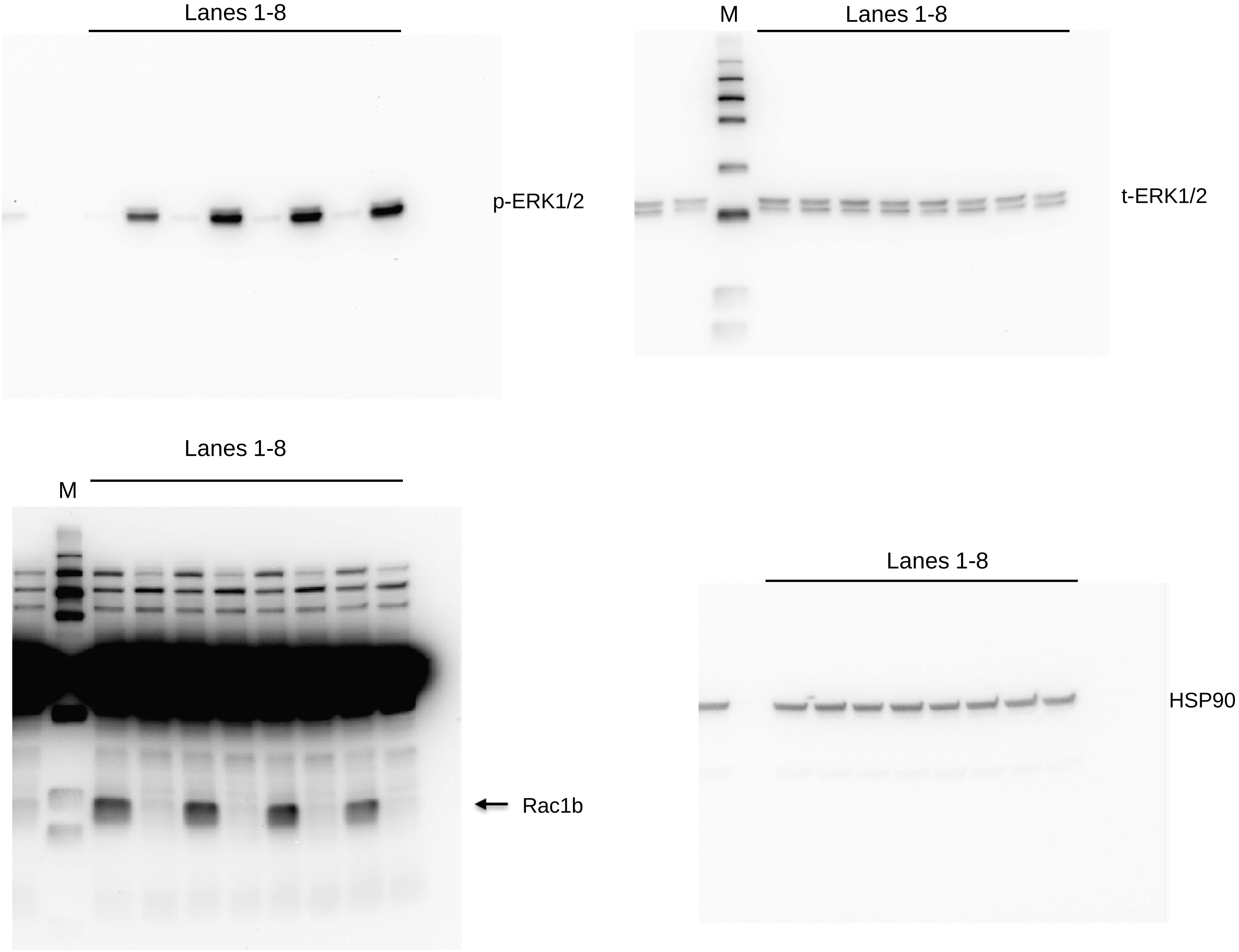

Lanes 1-8
p-ERK1/2
M
Lanes 1-8
t-ERK1/2
Lanes 1-8
M
Rac1b
Lanes 1-8
HSP90

Figure 2B

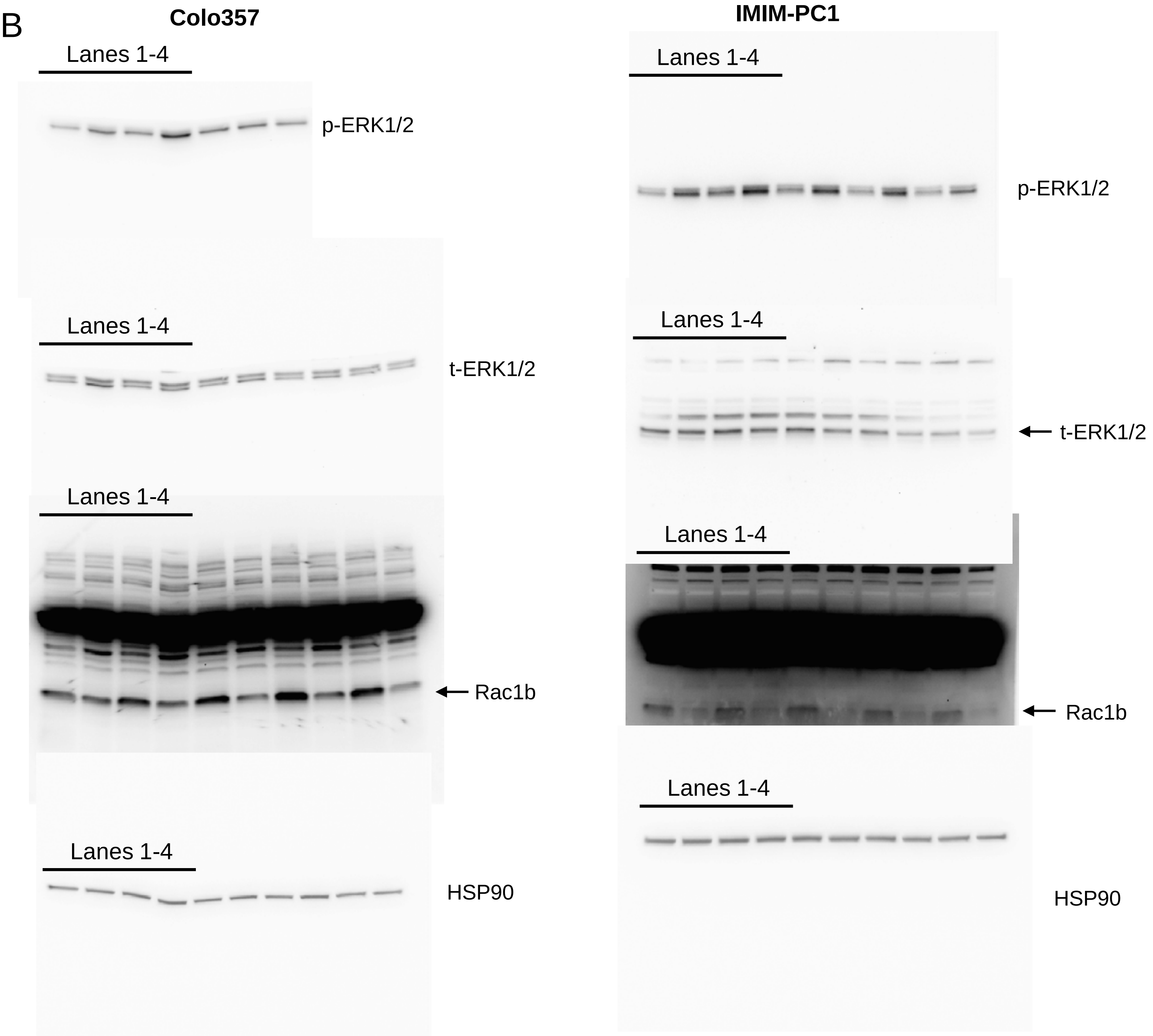
Colo357
IMIM-PC1
Lanes 1-4
p-ERK1/2
Lanes 1-4
p-ERK1/2
Lanes 1-4
t-ERK1/2
Lanes 1-4
t-ERK1/2
Lanes 1-4
Lanes 1-4
Rac1b
Rac1b
Lanes 1-4
Lanes 1-4
HSP90
HSP90

Figure 2C

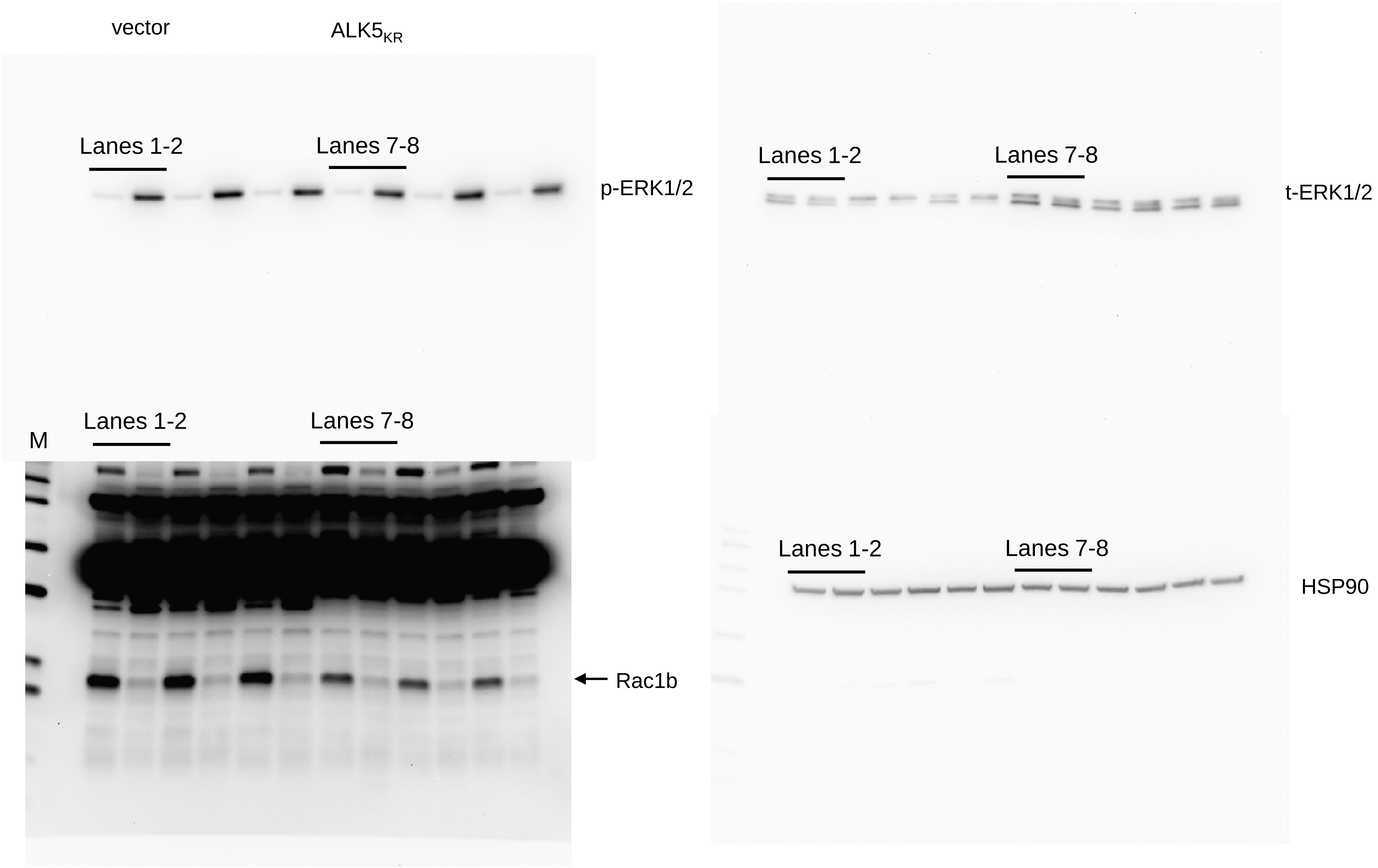
vector
ALK5KR
Lanes 1-2
Lanes 7-8
p-ERK1/2
Lanes 1-2
Lanes 7-8
t-ERK1/2
M
Lanes 1-2
Lanes 7-8
Rac1b
Lanes 1-2
Lanes 7-8
HSP90

Figure 2D

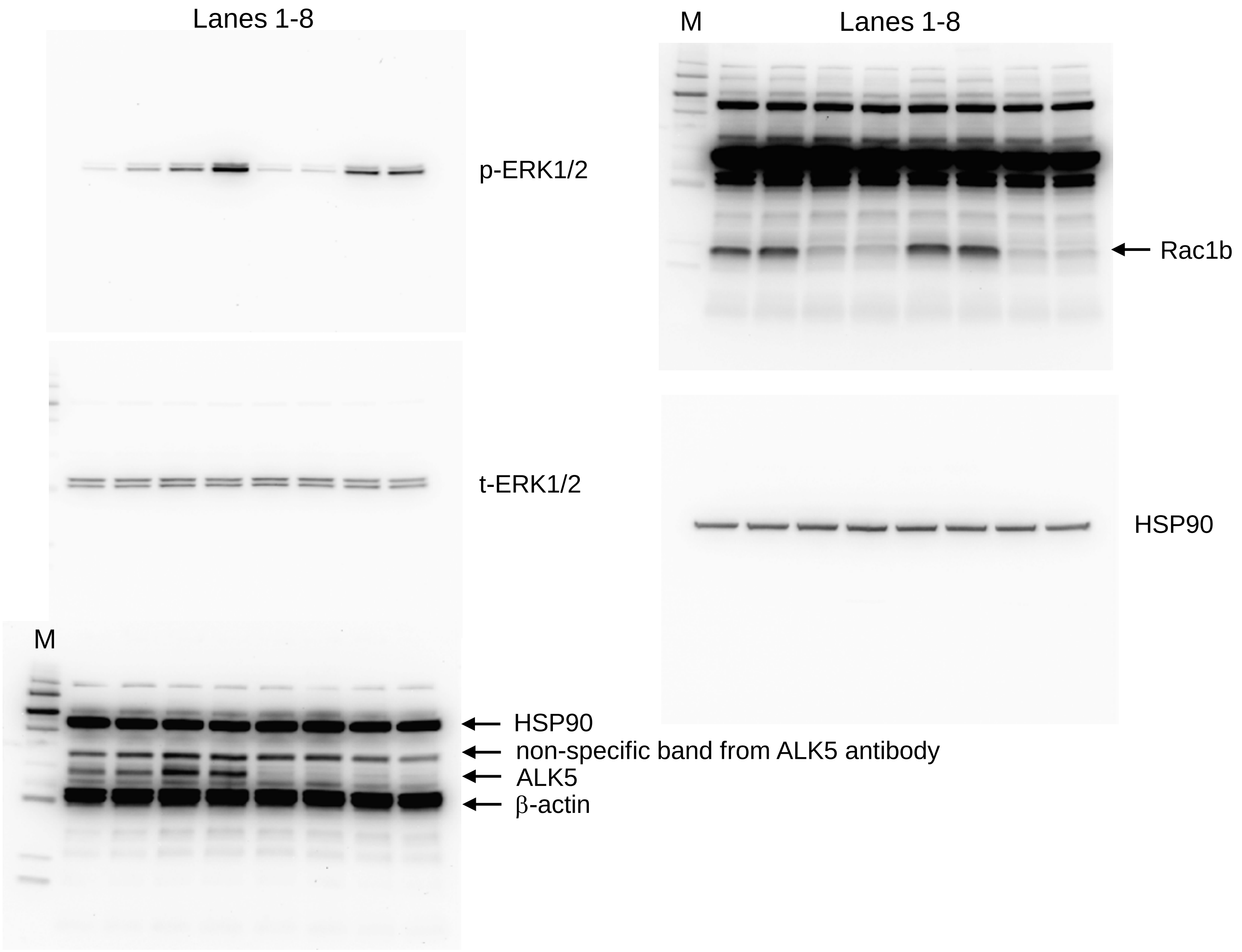
Lanes 1-8
p-ERK1/2
t-ERK1/2
M
HSP90
non-specific band from ALK5 antibody
ALK5
β-actin
M
Lanes 1-8
Rac1b
HSP90

Figure 3

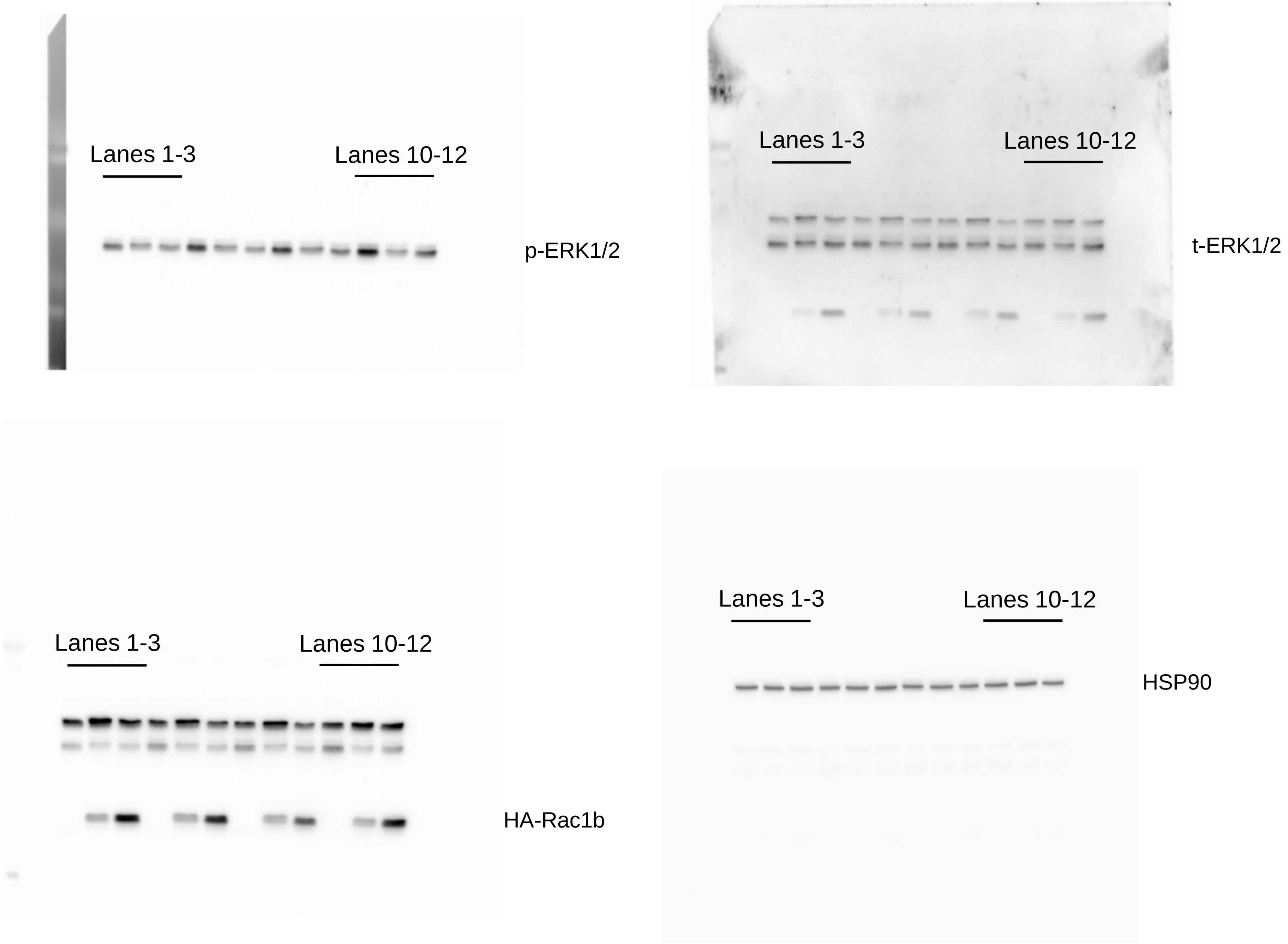

Lanes 1-3
Lanes 10-12
p-ERK1/2
Lanes 1-3
Lanes 10-12
t-ERK1/2
Lanes 1-3
Lanes 10-12
HA-Rac1b
Lanes 1-3
Lanes 10-12
HSP90

Figure 4B

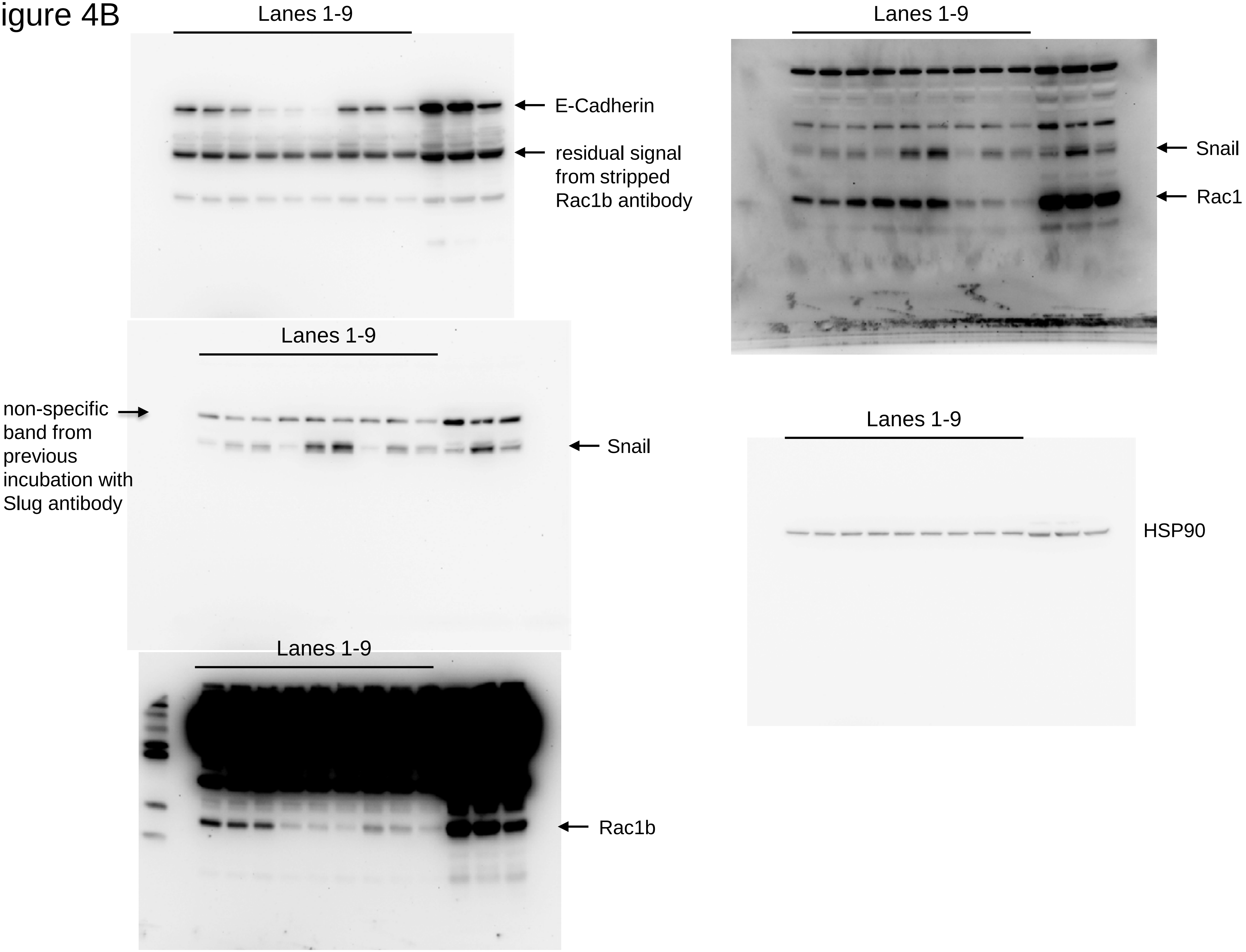

Lanes 1-9
E-Cadherin
residual signal from stripped Rac1b antibody
Lanes 1-9
Snail
Rac1
Lanes 1-9
non-specific band from previous incubation with Slug antibody
Snail
Lanes 1-9
HSP90
Lanes 1-9
Rac1b

Figure 6

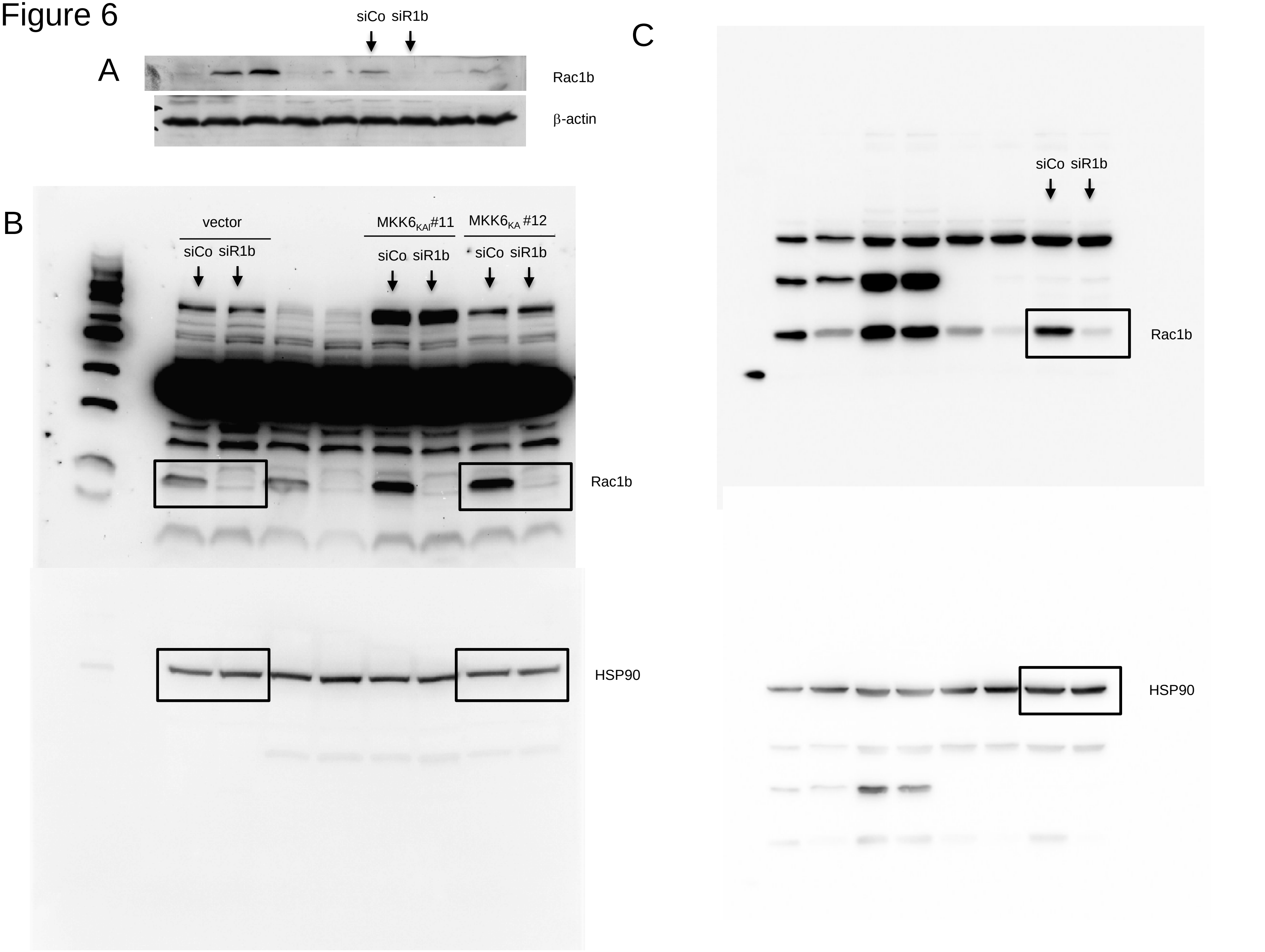

A
siCo siR1b
Rac1b
β-actin
B
vector
siCo siR1b
MKK6KAI#11
siCo siR1b
MKK6KA #12
siCo siR1b
Rac1b
HSP90
C
siCo siR1b
Rac1b
HSP90

Figure 6

D

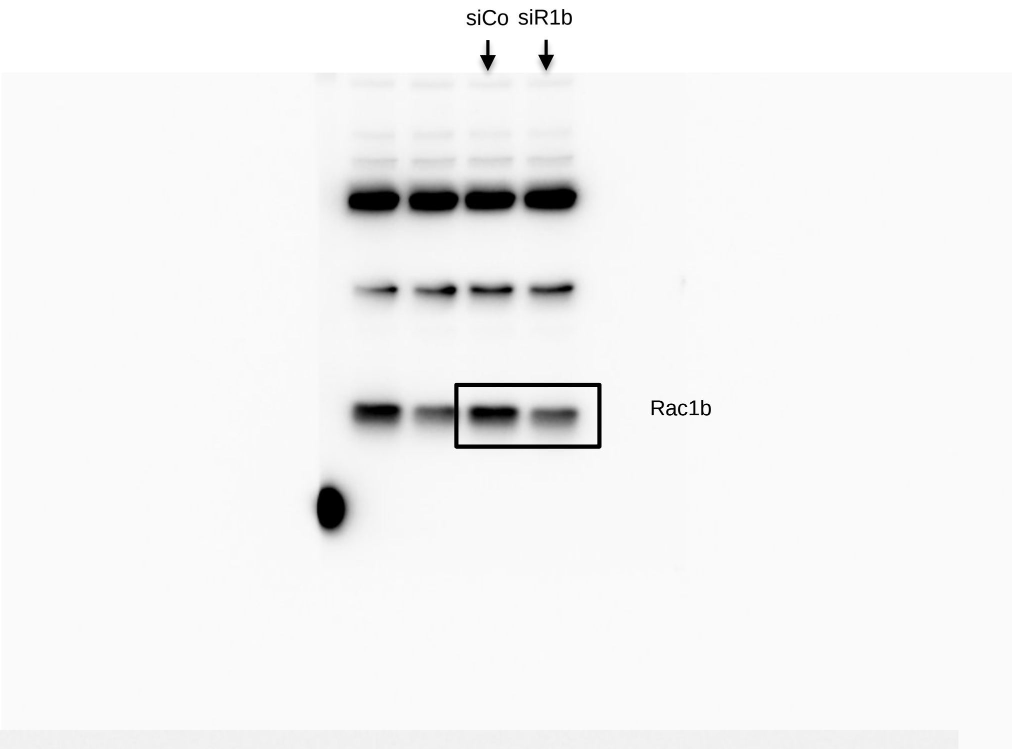

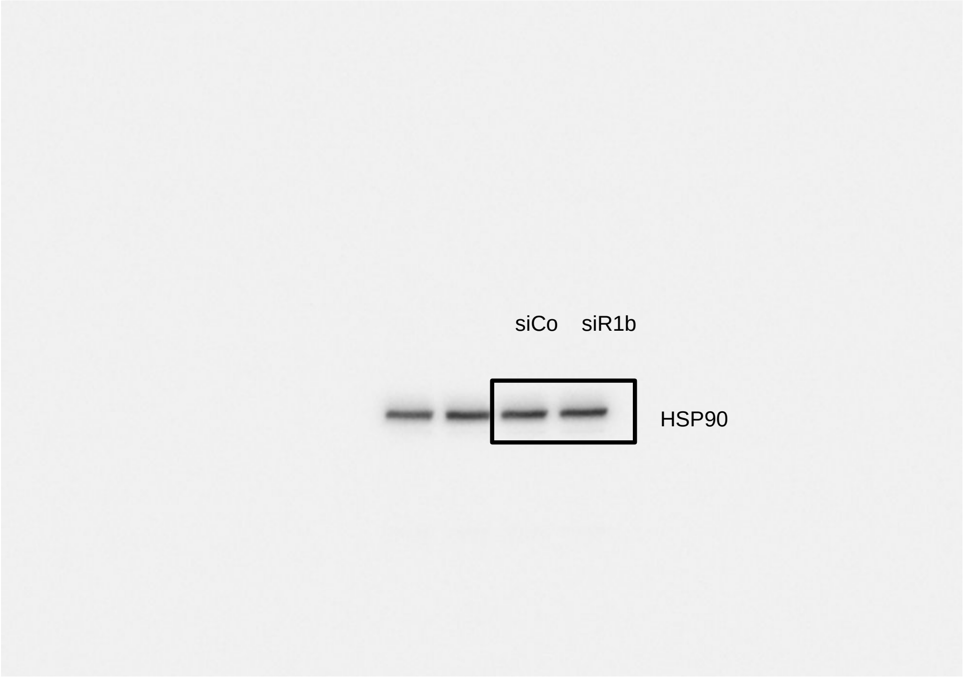

Supplementary Figure S1

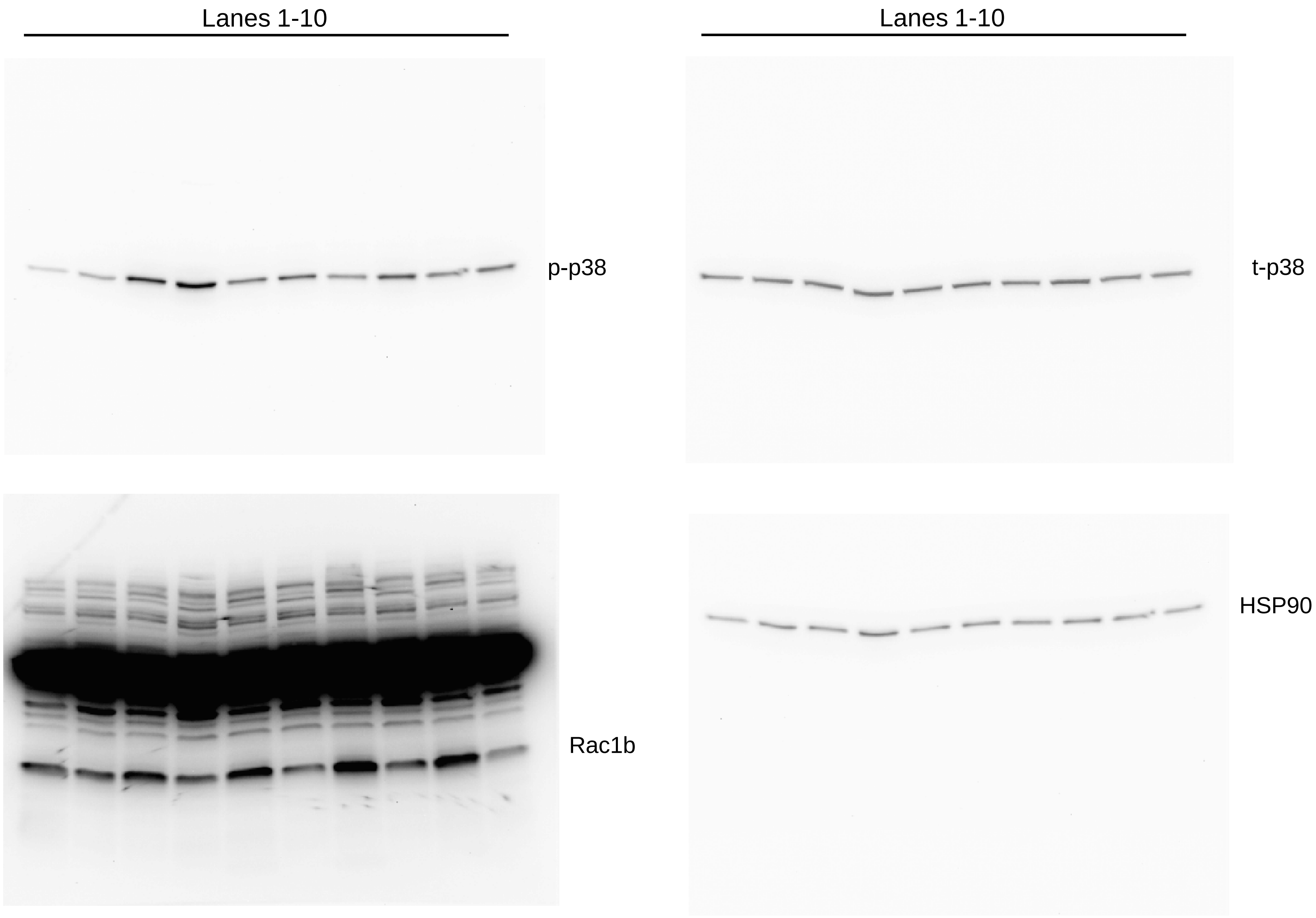

Supplementary Figure S2

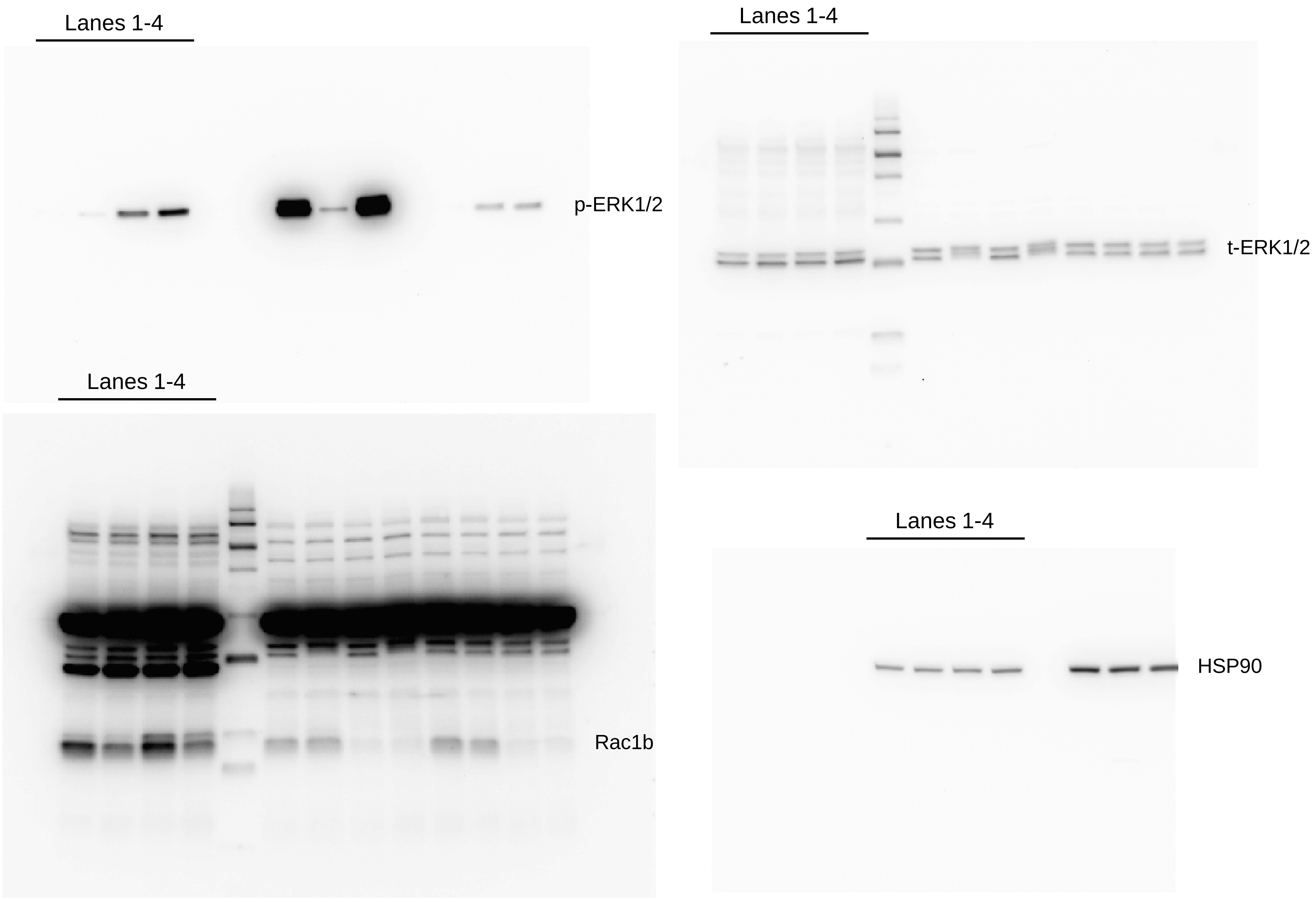

Supplementary Figure S3

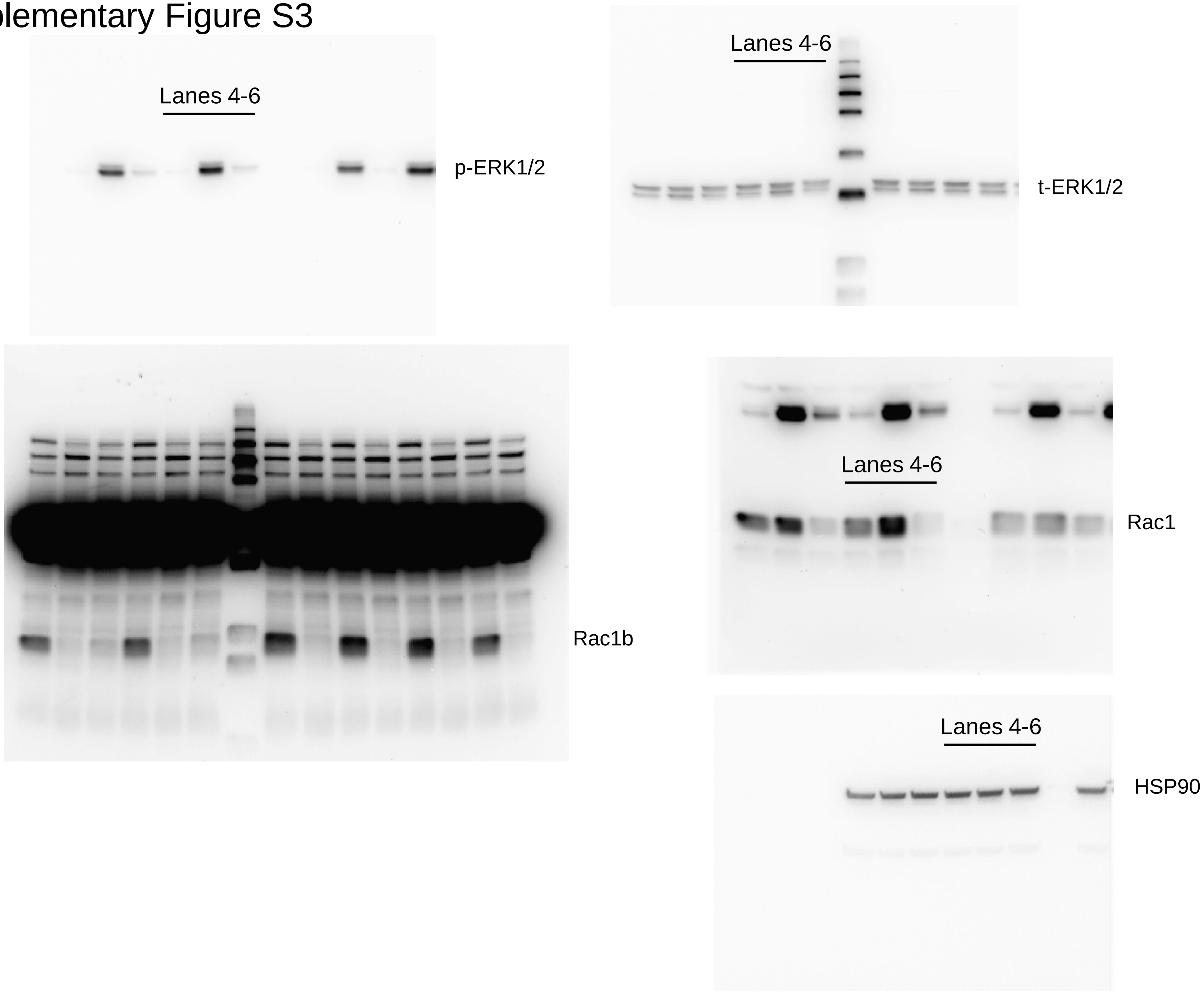

Supplementary Figure S4

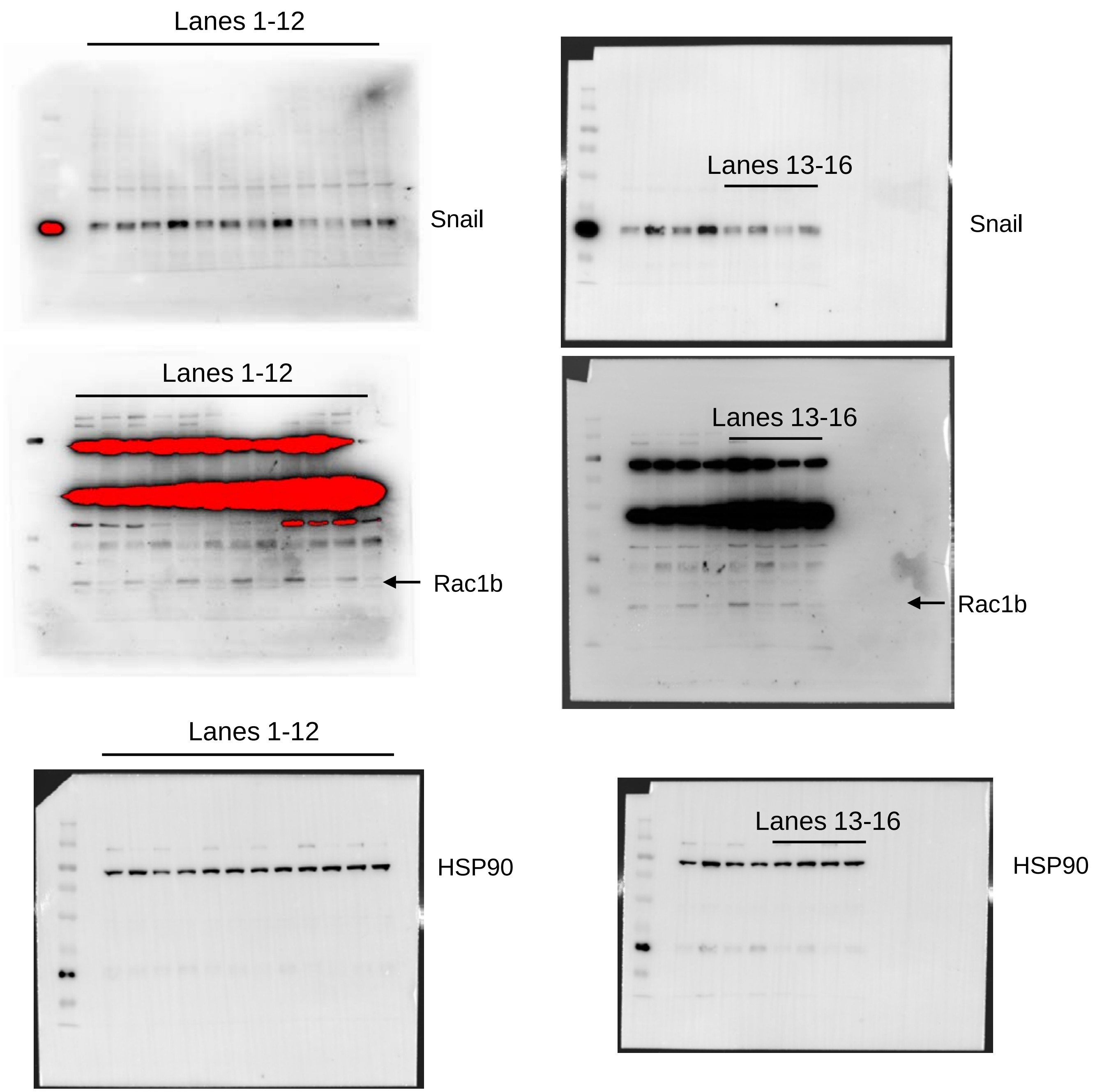

Supplement: Supplementary file 1 — Supplementary figures and tables [file 41598_2017_15170_MOESM1_ESM.pdf]
